# The most basal ankylosaurine dinosaur from the Albian–Cenomanian of China, with implications for the evolution of the tail club

**DOI:** 10.1038/s41598-018-21924-7

**Published:** 2018-02-27

**Authors:** Wenjie Zheng, Xingsheng Jin, Yoichi Azuma, Qiongying Wang, Kazunori Miyata, Xing Xu

**Affiliations:** 1 Zhejiang Museum of Natural History, Hangzhou, Zhejiang 310014 People’s Republic of China; 2 Key Laboratory of Vertebrate Evolution and Human Origins of Chinese Academy of Sciences, Institute of Vertebrate Paleontology and Paleoanthropology, Chinese Academy of Sciences, Beijing, 100044 People’s Republic of China; 3 CAS Center for Excellence in Life and Paleoenvironment, Beijing, 100044 People’s Republic of China; 4 University of Chinese Academy of Sciences, Beijing, 100049 People’s Republic of China; 5 State Key Laboratory of Palaeobiology and Stratigraphy (Nanjing Institute of Geology and Palaeontology, CAS, Nanjing, 210008 People’s Republic of China; 6 Institute of Dinosaur Research, Fukui Prefectural University, Fukui, 910-1195 Japan; 7 Fukui Prefectural Dinosaur Museum, Katsuyama, Fukui 911-8601 Japan; 8 Jinyun Museum, Jinyun, Zhejiang 321400 People’s Republic of China

## Abstract

The tail club knob is a highly specialized structure thought to characterize a subgroup of the ankylosaurine ankylosaurians, and the oldest documented tail club knob in the fossil record occurred in the Campanian ankylosaurine *Pinacosaurus*. Here we report a new ankylosaurid *Jinyunpelta sinensis*, gen. et sp. nov., from the Albian–Cenomanian Liangtoutang Formation, Jinyun County, Zhejiang, China. This is the first definitive and the best preserved ankylosaurid dinosaur ever found in southern China. *Jinyunpelta* possesses unique cranial features differs from other ankylosaurs including two paranasal apertures level with and posterior to the external naris, a triangular fossa on the anterodorsal edge of the maxilla, an antorbital fossa in the junction between the maxilla, lacrimal and jugal, and an anterior process of the prearticular that lies ventral to the splenial. Our phylogenetic analysis suggests *Jinyunpelta* as the most basal ankylosaurine dinosaur. *Jinyunpelta* has a tail club with interlocking caudal vertebrae and a well-developed tail club knob, it represents the oldest and the most basal ankylosaurian known to have a well-developed tail club knob. The new discovery thus demonstrates that a large and highly modified tail club evolved at the base of the ankylosaurine ankylosaurs at least about 100 million years ago.

## Introduction

Ankylosaurian fossils were first discovered in Huzhen Town, Jinyun County, Zhejiang Province in the 1970s, but they have never been formally described^1^. Most of these fossils were subsequently lost, though others were recently transferred to Zhejiang Museum of Natural History (ZMNH). In June of 2008, Mr. Meiyun Li, a local farmer, found a new ankylosaurian fossil in a construction site in Huzhen, Jinyun. Subsequently, a joint team from the Zhejiang Museum of Natural History, Jinyun Museum and Fukui Prefectural Dinosaur Museum organized several excavations at this site, and others, in Huzhen Town between 2008 and 2014. The 2013 excavation was particularly successful, producing more than five ankylosaurian individuals, though all incomplete. Here we describe two specimens that were collected during the 2013 fieldwork, as the other specimens are still under preparation.

## Geographical and Geological Settings

**Figure 1:**
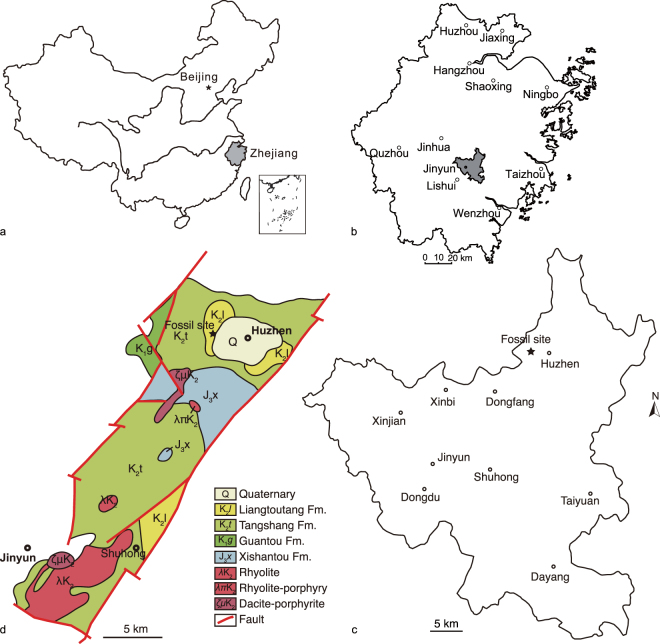
Locality and geological maps pertaining to the fossil locality. (**a**) The map of China showing Zhejiang Province. (**b**) The map of Zhejiang showing the Jinyun county; (**c**) map of Jinyun County with the fossil locality (marked by a star); (**d**) Geological map of Huzhen and Shuhong basins in Jinyun County showing type locality of *Jinyunpelta sinensis* (marked by a star), (**a**,**b**) and (**c**) Jin, *et al*.^46^, (**d**) after Lawver, *et al*.^47^.

Huzhen Basin is located between Xianju and Yongkang basins in eastern Zhejiang, covering about 300 km^2^ in area. The Huzhen basin outcrops the Cretaceous Tiantai Group, which consists of the Tangshang, Liangtoutang, and Chichengshan formations in ascending order (Fig. 1). Although the Liangtoutang Formation (also called the Laijia Formation) has been widely accepted as early Late Cretaceous^2^, some recent radiometric dates suggest that the Liangtoutang Formation is late Early Cretaceous to early Late Cretaceous (Albian–Cenomanian). For example, Wang, *et al*.^3^ reported a radiometric date of 105.9–103.2 Ma (Albian) for the Liangtoutang Formation; He, *et al*.^4^ reported a SIMS zircon U–Pb date of about 99–96 Ma (Cenomanian) for the Liangtoutang (Laijia) Formation in Tiantai basin; Jiang, *et al*.^5^ reported a zircon U-Pb age of 100 ± 1 Ma (Albian–Cenomanian) for the Liangtoutang (Laijia) Formation in Tiantai. Taking into account all available radiometric dates and stratigraphic correlation, we place the Liangtoutang Formation in the mid-Cretaceous, Albian–Cenomanian (105.9–96 Ma).

## Results

**Systematic palaeontology**

Dinosauria Owen 1842

Ornithischia Seeley, 1888

Thyreophora Nopcsa, 1915

Eurypoda Sereno, 1986

Ankylosauria Osborn, 1923

Ankylosauridae Brown, 1908

Ankylosaurinae Brown, 1908

*Jinyunpelta sinensis* gen. et sp. nov.

Etymology: The generic name derives from ‘Jinyun’ (Mandarin) in reference to Jinyun County in which the type locality is located, and ‘pelta’ (Latin), a small shield, in reference to the osteoderms found on all ankylosaurians. The root of the specific name ‘sin’ (Greek) refers to China, the country of origin.

Holotype: ZMNH M8960, an almost complete skull, and a partial postcranial skeleton including some cervical, dorsal and sacral vertebrae, partial tail club knob, dorsal ribs, right scapula, partial right manus, left ilium, both ischia, left femur, osteoderms and numerous small postcranial ossicles.

Paratype: ZMNH M8963, A partial postcranial skeleton including an almost complete tail club, left tibia and fibula.

Locality: Lijin Industrial Park, Huzhen Town, Jinyun County, Zhejiang Province, China (Fig. 1).

Stratigraphic horizon: Liangtoutang Formation, later Early to early Late Cretaceous (Albian–Cenomanian).

Diagnosis: A derived ankylosaurid dinosaur differing from other ankylosaurid species in having the following combinations of features (autapomorphies indicated by *): two paranasal apertures C_1_ and C_2_ located posterior to the external naris and the center of the apertures on the same level with the center of the external naris*; a triangular fossa on anterodorsal edge of the maxilla*; two oval cavities on the dorsal of the nasal; the antorbital fossa present in the junction area of the maxilla, lacrimal and jugal; the prefrontal extending ventrally and contacts the maxilla; the postorbital excluded from the posterior rim of the orbit*; the anterior portion of the prearticular underlying the posterior portion of the splenial*; the dorsal centrum elongated with the ratio of length to the width more than 1.3; the tail club knob roughly hexagonal in dorsal view, with the widest point close to the distal end; and a prominent scar present mediodorsal to the medial condyle of the femur*.

### Description and comparison

The ZMNH M8960 and ZMNH M8963 were discovered in the same quarry approximately two to three meters apart.

### Skull

**Figure 2:**
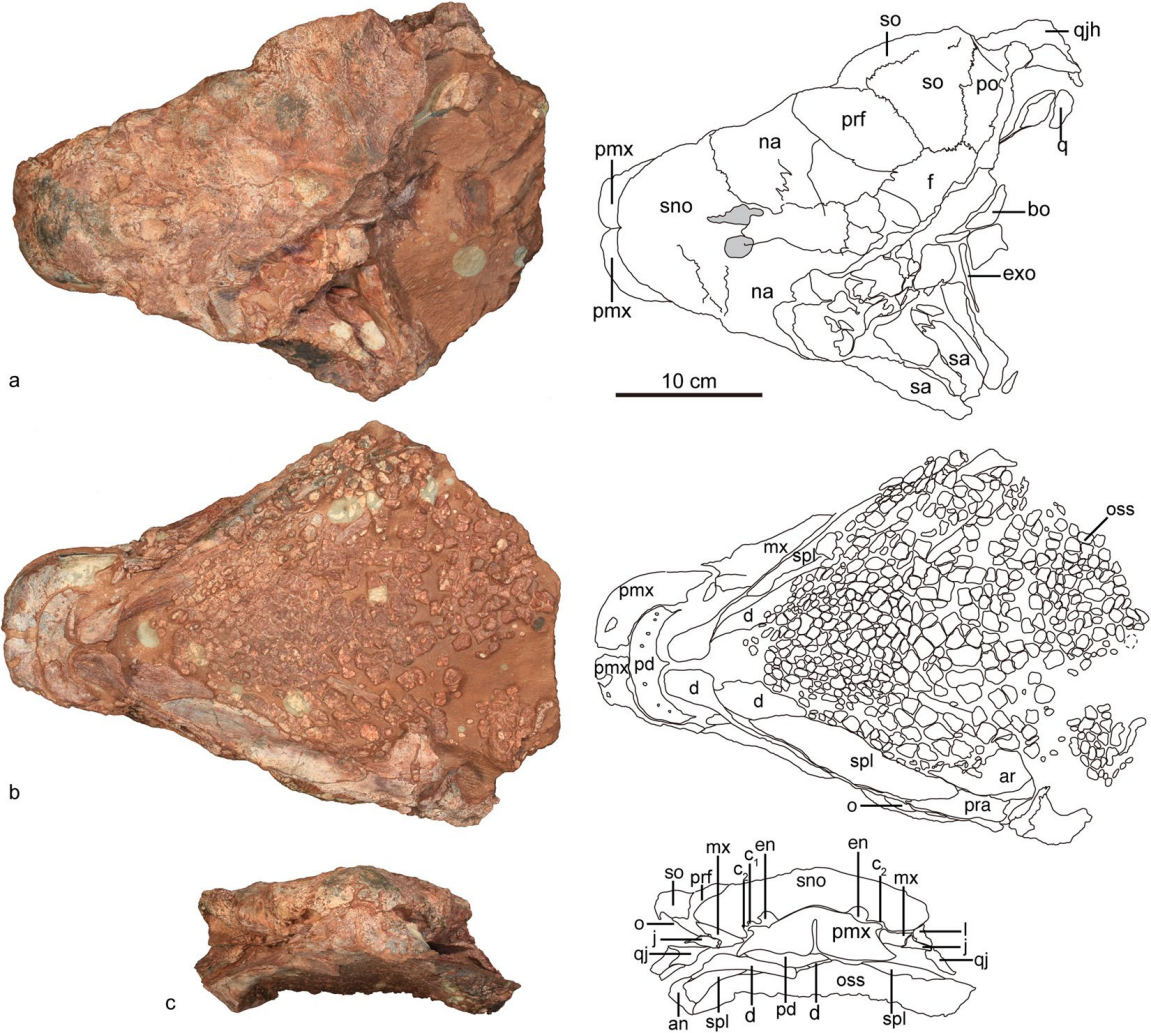
The skull and mandible of *Jinyunpelta sinensis* holotype ZMNH M8960. Photograph and line drawing of the skull and mandible in dorsal (**a**), ventral (**b**), and anterior (**c**) views. Abbreviations: an, angular; ar, articular; b, paranasal aperture B; bo, basioccipital; c_1_, paranasal aperture C_1_; c_2_, paranasal aperture C_2_; d, dentary; en, external naris; exo, exoccipital; f, frontal; fen, fenestra; l, lacrimal; mso, middle supraorbital; mx, maxilla; mf, maxillary fossa; na, nasal; o, orbit; oss, ossicle; pd, predentary; pmx, premaxilla; po, postorbital; pra, prearticular; prf, prefrontal; q, quadrate; qj, quadratojugal; qjh, quadratojugal horn; sa, surangular; sno, supranarial ornamentation; spl, splenial; sq, squamosal.

**Figure 3:**
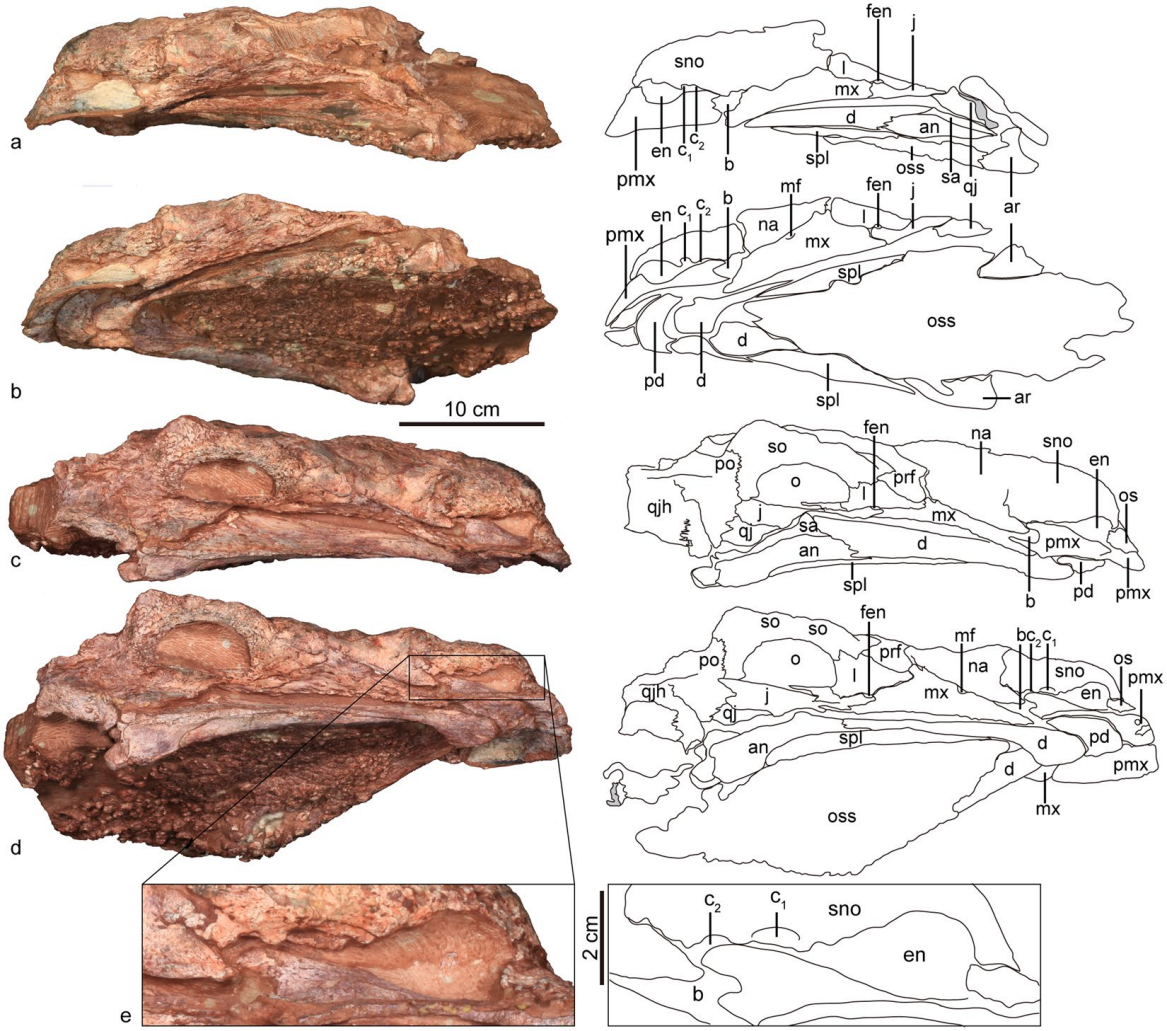
The skull and mandible of *Jinyunpelta sinensis* holotype ZMNH M8960. Photograph and line drawing of the skull and mandible in left lateral (**a**), left ventrolateral (**b**), right lateral (**c**) and right ventrolateral (**d**) views, and enlarged view of the right narial portion (**e**). Abbreviations: an, angular; ar, articular; b, paranasal aperture B; bo, basioccipital; c_1_, paranasal aperture C_1_; c_2_, paranasal aperture C_2_; d, dentary; en, external naris; exo, exoccipital; f, frontal; fen, fenestra; l, lacrimal; mso, middle supraorbital; mx, maxilla; mf, maxillary fossa; na, nasal; o, orbit; qjh, quadratojugal horn; oss, ossicle; pd, predentary; pmx, premaxilla; po, postorbital; pra, prearticular; prf, prefrontal; q, quadrate; qj, quadratojugal; qjh, quadratojugal horn; sa, surangular; sno, supranarial ornamentation; spl, splenial; sq, squamosal.

The skull and mandible of ZMNH M8960 are preserved together, with the dorsoposterior portion damaged, such that the squamosals and the posterior-most part of the frontals, postorbitals, parietals and occipital region are missing (Figs 2, 3, Table 1). The lateral sides of the snout taper anteriorly towards a squared-off premaxillary beak and there is a constriction anterior to the orbit in dorsal view. The twisted maxillary and dentary tooth row are strongly inset medially. Gular ossicles of the lower jaw obscure the palate.

**Table 1:** Measurements of the skull of *Jinyunpelta sinensis* holotype ZMNH M8960.

	Element	Measurement (cm)
Skull dimension	Width across supraorbitals	27.0 (estimated)
	Total length	33.5 (preserved portion)
Orbit dimension	Anteroposterior width	6.2
	Height	3.3
Lower jaw	Length of lower jaw	28.8
Predentary	Mediolateral Width of predentary	8.1
	Dorsoventral Height	3.0
Right maxilla	Anteroposterior length	12.7
	Height	3.1

The skull is longer than wide, unlike the wider skulls in derived ankylosaurids^6^. Some sutures of the dorsum posterior to the nasals are not fully fused. The sutural contacts of the skull are known from only a few taxa: *Pinacosaurus grangeri* from China and Mongolia, *P. mephistocephalus*, *Liaoningosaurus* and *Chuanqilong* from China, *Kunbarrasaurus* from Australia and *Cedarpelta* from the United States^7,8^. The dorsal surface of the skull has irregular, roughly textured skull ornamentation with some randomly directed non-vascular grooves, as in basal ankylosaurids such as *Gobisaurus*, *Shamosaurus*, *Pinacosaurus* and ‘*Zhongyuansaurus*’, rather than discrete caputegulae as in more derived ankylosaurids (Ankylosaurinae) like *Ankylosaurus*, *Euoplocephalus* or *Saichania*^6^. In lateral view, the skull bears a nearly flat dorsal surface posterior to the nares with only slight arching of the maxillary rostrum, as in ‘*Zhongyuansaurus*’^9^, and *Kunbarrasaurus*^8^. In contrast, *Gobisaurus* exhibits a domed dorsal surface^10^. The flatness of the skull may also partially result from taphonomic crushing.

#### Antorbital fossa

The junction of the maxilla, lacrimal and jugal possesses a fossa, clearly present on both sides of the skull (Figs 2, 3). This probably represents a reduced antorbital fossa. A small antorbital fossa is similarly present between the maxilla and lacrimal in juvenile *Pinacosaurus*^11^, but an antorbital fenestra/fossa is absent in most ankylosaurians^7^.

#### External naris

The internarial bar, a medially positioned extension of the premaxilla that arcs posterodorsally, separates the external nares from one another (Fig. 2). The external naris angles anterolaterally to be visible in anterior view, unlike the anteriorly directed external naris in *Crichtonpelta*^12^. There are three paranasal apertures posterior to the external naris and are marked as opening B, C_1_ and C_2_ according to Hill, *et al*.^13^ (Fig. 3). Opening B sits between the premaxilla and maxilla, whereas the opening is fully located within the premaxilla in *Pinacosaurus*^13,14^. Opening B remains relatively small compared with that of *Pinacosaurus*^13,14^. Apertures C were considered to be the openings into a relatively large sinus system within the premaxilla^13,14^. The openings C_1_ and C_2_ sit posterior to the external naris, the center of the apertures is on the same level with the center of the external naris (Fig. 3). Although apertures C in *Pinacosaurus* are variable in number, the center is positioned much lower that external naris^13,14^. The intranasal process, partitioning of the external naris and paranasal aperture, directs dorsoposteriorly. The cranial ornamentation is not partitioned with amorphous texture. Prominent furrow ornamentations occur on the anterior surface of the nasal immediately posterodorsal to the external naris.

#### Orbit

The subcircular orbits face anterolaterally (Figs 2, 3). Dorsoventral compaction during fossilization has shortened the dorsoventral diameter of the orbit. The orbits are visible in anterior view because of the anterior tapering of the skull as in *Ankylosaurus*^15^ and *Crichtonpelta*^12,16^. The orbital edge is everted, as in *Saichania*, *Tarchia*^14^, and *Crichtonpelta*^16^.

The lacrimal incisure is absent as in *Gobisaurus*^10^, and *Crichtonpelta*^16^, in contrast, the incisure is present in *Pinacosaurus grangeri* and *Tarchia* (INBR21004)^15,17^.

### Rostral region

#### Premaxilla

The premaxillae form a broad, sub-quadrate, edentulous beak, and bound the paranasal apertures and the external naris ventrally (Fig. 2). The beak is also broad in ankylosaurines such as *Ankylosaurus*^15^, *Pinacosaurus*^13,18^ and *Euoplocephalus*^19^. In contrast, the beak is much narrower in *Shamosaurus*^20^, *Gobisaurus*^10^, and *Crichtonpelta*^16^. The tomial crest formed by premaxillae is variably scalloped, and its outer borders curve downward, forming a sharp cutting edge.

The middle joint of the contralateral premaxillae is unfused and the suture is clearly visible in anterior view. A deep inverted V-shape premaxillary notch incises the interpremaxillary suture at its most anteroventral point, as in other ankylosaurids^7^. There are fused osteoderms on the dorsal surface of premaxilla. However, osteoderms are absent on the premaxilla in most ankylosaurids, such as *Pinacosaurus*^13,18^ and *Euoplocephalus*^19^.

In palatal view, the interpremaxillary suture is also clearly visible. The premaxillary palate is deeply concave, sub-quadrate, wider than long, parallel-sided, and only slightly rounded at its anterior edge. A posterodorsally inclined parasagittal incisive foramen sits directly posterior to the premaxillary notch along the palatal surface of each premaxilla. The posterior portion of the premaxilla is obscured by the lower jaws. Dorsolaterally, the sutural contact with adjacent nasals is obscured. The premaxilla-maxilla suture inclines backwards, unlike the vertically oriented ventral portion of premaxilla-maxilla suture in *Pinacosaurus*^14^.

#### Maxilla

In lateral view, the maxilla has a low triangular outline (Fig. 3). Anterodorsally, the maxilla contacts the premaxilla and nasal by a straight suture. The contact with the premaxilla is restricted to anterodorsal edge of the anterior tip of the maxilla, whereas most of the anterodorsal edge is in contact with the nasal. A fossa (Fig. 3b,d: mf) occurs immediately posteroventral to the middle of the anterodorsal edge. The fossa, present on both maxillae, is triangular with the anteroventral edge paralleling the posterodorsal edge of the maxilla. Anteriorly the maxilla contributes to the posterior margin of the paranasal opening B, which is formed by the maxilla, premaxilla and nasal.

The maxilla contacts the prefrontal, lacrimal and jugal posterodorsally. The maxilla has a posterodorsal process meeting the prefrontal. The contact between the maxilla and the lacrimal is long. The most ventral portion of the posterodorsal edge of the maxilla represents the jugal contact.

The roughened lateral surface of the maxilla bears scattered foramina. A co-ossified osteoderm laterally overlays the maxillary portion of the tomial crest, similar to *Pinacosaurus*^11^, but unlike adult specimens of *Euoplocephalus*^11,19^. The lateral surface is flat, unlike the convex surface in *Crichtonpelta*^16^.

The tooth row is inset medially from the lateral side of the maxilla as is typical for ankylosaurs^11^, but remains invisible in lateral view in this specimen due to the dorsoventral compression.

#### Nasal

The anterior half of the nasals appear highly sculptured and rugose and sculpturing obscures the median sagittal suture between the nasals. The ornamentation is irregular, similar to *Gobisaurus*^10^, *Shamosaurus*^20^, and *Crichtonpelta*^16^. The nasal extends far backward along the skull roof. Posteriorly, the nasal contacts the frontal in line with the midpoint of the orbit. The serrated suture with the frontal curves anteromedially, unlike the roughly transversely oriented suture in *Pinacosaurus*^14^, and *Kunbarrasaurus*^8^. The nasal contacts the prefrontal posterolaterally, and the long contact is oriented lateroanteriorly, and passes from the dorsal to the lateral side of the skull. The nasal is widest at the anterolateral end of the suture, and becomes narrower toward both anterior and posterior ends, producing a hexagon shape in dorsal view. Dorsally, the nasal bears two oval cavities, symmetrical with each other sagittally. The nasal contributes to a large portion of the preorbital region forming the lateral wall of the skull. Ventrally, the nasal contacts the anterodorsal edge of the maxilla, similar to *Minmi*^21^. The contact between the nasal and the maxilla is relatively shorter in *Pinacosaurus*^14^. The nasal lacks a contact with the lacrimal, unlike in *Pinacosaurus*^14^.

#### Prefrontal

The prefrontal sits posterolateral to the nasal, anterolateral to the frontal, anteromedial to the supraorbital, and dorsal to the lacrimal. In dorsal view, the prefrontal is oval-shaped, orienting anterolaterally with respect to the sagittal plane. The relatively large prefrontal contributes to the dorsal and lateral walls of the skull. Posteromedially its contact with the frontal is short and its contribution to the lateral wall of the skull is insignificant, with a small rectangular lateral exposure at the rostral region of the orbit as in *Pinacosaurus*^14^. The prefrontal contacts the maxilla anteroventrally. In contrast, the lacrimal separates the prefrontal from the maxilla in *Pinacosaurus*^11,14^. The prefrontal of *Kunbarrasaurus* seems to contact the dorsally strongly expanded maxilla^8^.

#### Lacrimal

In lateral view, the lacrimal is triangular and tapers anteriorly and forms the anterior margin of the orbit (Fig. 3). Anteroventrally, the lacrimal contacts the maxilla along a suture that directs obliquely upward. Dorsally the lacrimal contacts the prefrontal and supraorbital; and posteroventrally the lacrimal contacts the jugal below the orbit. The lacrimal is rectangular in juvenile *Pinacosaurus*^22^.

### Temporal region

#### Supraorbitals

Two supraorbitals are evidently incorporated into the skull roof, but their contacts with neighboring bones are not fully discernible. They form the upper orbital margin and protrude laterally over the orbit as wedge-like bosses. They form a blunt horn near the lateroposterior end of the skull. The horn is prominent in lateral view but inconspicuous in dorsal view. They are rounded and blunt as in *Gobisaurus*^10^, and *Shamosaurus*^6,20^. In dorsal view, the two supraorbital caputegulae form a continuous lateral edge as in *Ankylosaurus*, *Anodontosaurus*, *Euoplocephalus*, *Dyoplosaurus*, and *Scolosaurus*. In contrast, the supraorbital caputegulae have distinct peaks in *Pinacosaurus*^14^, *Tarchia*, *Ziapelta*^23^, and *Zuul*^22^. The supraorbital horn is well separated from the squamosal horn as in *Crichtonpelta*^16^, *Anodontosaurus*, *Euoplocephalus*, and *Scolosaurus*, however the supraorbital horn is continuous with the squamosal horn in *Ankylosaurus*^24^.

The elongate anterior supraorbital seems to contribute more to the lateral margin of the orbit than the posterior supraorbital. The posterior supraorbital is triangular and larger than the anterior supraorbital. The former is located medially to the latter. The anterior supraorbital contacts the lacrimal and the prefrontal. The posterior one contacts the prefrontal, the frontal and the postorbital. Besides the anterior and posterior supraorbitals, a third supraorbital is present in juvenile *Pinacosaurus*^13^, and many small pyramidal caputegulae in *Zuul*^22^.

#### Postorbital

The postorbital forms part of the posterolateral corner of the skull, posterior to the posterior supraorbital. The horizontal wing is incorporated into the skull roof and contacts the supraorbital and frontal anteriorly. The vertical wing of the postorbital is excluded from the posterior part of the orbit, but contacts the jugal ventrally. In contrast, the vertical wing of the postorbital forms the posterior part of the orbit in *Pinacosaurus*^14^ and *Kunbarrasaurus*^8^.

#### Jugal

The jugal forms the ventral border of the orbit, and the suborbital arch is shallow as other ankylosaurs^19,25^. Ventrally the jugal displays a short, tapering continuance of the buccal emargination. The jugal articulates with the lacrimal anteriorly, the maxilla anteroventrally, the postorbital posterodorsally, and the quadratojugal posteriorly. The tapering anterior end of the jugal inserts between the posterior ends of the maxilla and lacrimal.

#### Frontal

Only the anterolateral portion of the right frontal is preserved and an osteoderm partially covers the naso-frontal suture. The frontal contacts the prefrontal anterolaterally, the supraorbital laterally, and the postorbital posterolaterally. The sutures are serrated, except the medial suture with the nasal.

#### Quadratojugal

Only the base portion of the right quadratojugal horn is preserved. The quadratojugal projection extends lateroventrally and bears shallow, irregular furrows on the external surface of the horn.

### Mandible

The associated mandible lacks an external mandibular fenestra. Additionally, there is not a well-developed mandibular caputegulum, but its ventral surface is sculptured as in juvenile *Pinacosaurus*. This indicates the ornamentation probably represents sculpturing and outgrowth of the bones themselves, rather than being produced by osteoderms, as suggested by Arbour and Evans^22^.

#### Predentary

The predentary is transversely wide and crescentic in ventral view. The predentary conjoins the paired dentaries anterior to the mandibular symphysis, forming an edentulous tomium. A short, ventrally projecting sagittal protuberance of the predentary contacts the mandibular symphysis. Externally the anteroventral surface is rugose and perforated by a variety of foramina.

#### Dentary

Gular ossicles obscure the tooth row. Anteriorly, the dentaries flex medially and articulate with one another at the dorsoventrally abbreviated mandibular symphysis. Dorsal to the symphysis, the dentaries articulate with the predentary. In lateral view, the dentary articulates with the surangular posteriorly, the angular posteroventrally, and the predentary anteriorly. In lingual view, the dentary is mostly overlain by the splenial.

#### Splenial

The splenial forms the ventromedial surface of the mandible. Posteriorly, the splenial contacts the prearticular, with its posterior end underlain by the prearticular. In contrast, the splenial underlies the anteroventral margin of the prearticular in *Euoplocephalus*^19^, *Tarchia* (Observation based on AMNH 31765, the cast of the ‘*Minotaurasaurus ramachandrani*’ holotype INBR21004)^17,26^ and *Zuul*^22^.

#### Angular

The angular forms the ventrolateral margin of the mandible, and as in most ankylosaurs, its lateral surface is invariably embellished with a rugose ornamentation. The angular articulates with the dentary anterodorsally, and the surangular dorsally. The ventral surface of the angular is sculptured but lacks the prominent ornamentation that occurs in subadult *Pinacosaurus* individuals^7,13^. The anterior process is short and similar to *Pinacosaurus*^14^, in contrast, the angular is elongate in *Euoplocephalus*^19^. The angular is not visible in medial view like in *Edmontonia*, in contrast with *Euoplocephalus*^19^.

#### Surangular

The surangular is situated in the dorsoposterior quarter of the mandible where it articulates with the dentary anteriorly and angular ventrally. There are some small ossicles preserved on the lateral surface of the surangular.

#### Prearticular

The prearticular articulates with the splenial anterodorsally and the articular dorsally.

#### Articular

The articular overlies the splenial and the prearticular in the medial view.

#### Hyobranchium

Two rodlike ceratobranchial bones are preserved within the skull, but are mostly covered by gular ossicles. The hyoid bones display longitudinal striae.

#### Gular ossicle

Numerous osteoderms from 5 to 15 millimeter in diameter (gular ossicle) are preserved on the ventral side of the skull (Fig. 2). Their preservation is very similar to that in the holotype of *Panoplosaurus* (CMN 2759)^27^. Gular ossicles are also known in *Edmontonia* (AMNH 5381)^28^ and *Zuul*^22^. The ossicles near the mandible are arranged parallel with the mandible, while the remainder of the osteoderms have a random arrangement. The ossicles in the central area are relatively larger and the anterolateral and posterior ossicles are relatively small. This arrangement pattern is similar to *Panoplosaurus* (CMN 2759)^27^.

### Axial skeleton

#### Cervical vertebrae

**Figure 4:**
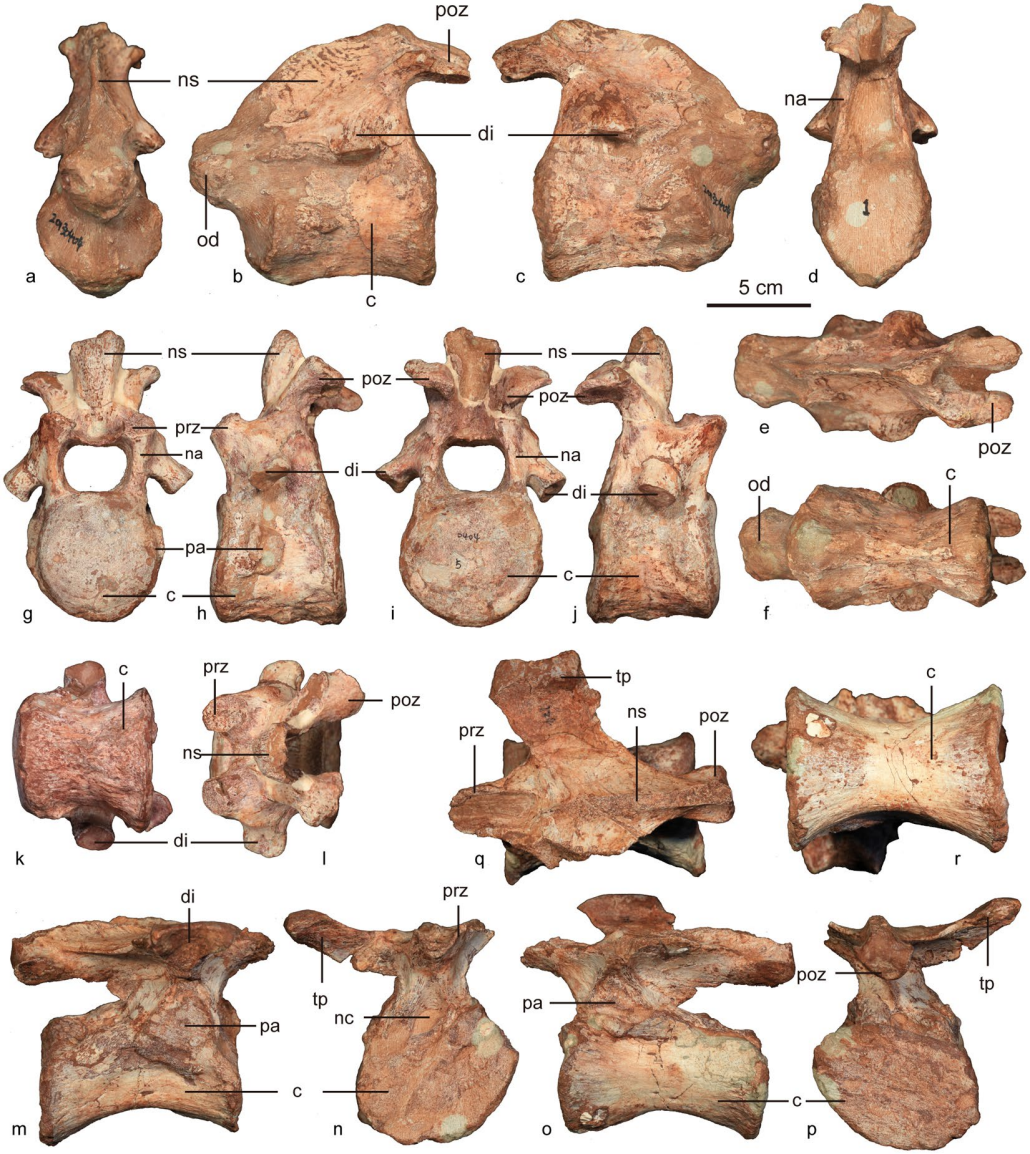
The vertebrae of *Jinyunpelta sinensis* holotype ZMNH M8960. The axis in anterior (**a**), left lateral (**b**), right lateral (**c**), posterior (**d**), dorsal (**e**) and ventral (**f**) views; the postaxial cervical vertebra in anterior (**g**), left lateral (**h**), posterior (**i**), right lateral (**j**), ventral (**k**) and dorsal (**l**) views; the dorsal vertebra in right lateral (**m**), anterior (**n)**, left lateral (**o**), posterior (**p**), dorsal (**q**) and ventral (**r**) views. Abbreviations: c, centrum; di, diapophysis, na, neural arch; nc, neural canal; ns, neural spine; od, odontoid process; pa, parapophysis; poz, postzygapophysis; prz, prezygapophysis; tp, transverse process.

The holotype ZMNH M8960 includes the axis (Fig. 4a–f) and one posterior cervical (Fig. 4g–l) (Table 2).

**Table 2:** Measurements of the vertebrae of *Jinyunpelta sinensis* holotype ZMNH M8960 (cm). Asterisk indicates incomplete measurement due to damage.

	Length of centrum	Width of anterior surface of centrum	Height of anterior surface of centrum	Minimum Transverse Width of Centrum	Height of posterior surface of centrum	Width of posterior surface of centrum	Width of neural canal	Height of neural canal
Axis	9.2	5.6*	—	3.2	6.1*	4.9	—	—
cervical vertebra	6.1	5.7	6.0	4.5	6.1	6.5	3.2	2.7
dorsal vertebra	9.7	7,7	5.3	4.3	5.3	8.1	1.5	1.3

The laterally compressed axial centrum has a concave posterior articulation surface, and is anteroposteriorly longer than the cervical centra in ZMNH M8960. The axis bears a robust neural arch with a short neural spine and a wide and massive dens. The neural spine slopes dorsoposteriorly and the dorsal edge is convex dorsally. The postzygapophyses are located on the posterolateral ends of neural spine, and exceed the posterior end of the centrum. They are almost parallel with each other in dorsal view and are directed ventroposteriorly in lateral view. The neural canal is deep and laterally compressed with an oval outline.

One postaxial cervical vertebra is well preserved in ZMNH M8960. The cervical centrum is wider than long, with subcircular amphicoelous articular faces. The pre- and postzygapophysis are elongate, extending slightly beyond the articular surfaces of the centrum. There is no epipophysis. The neural canal is wider than high, the dorsal and ventral edges are straight and the lateral edges are round. The transverse process (diapophysis) is located anteroposteriorly centrally and relatively low on the neural arch. The process projects ventrolaterally about 45° from the horizontal. In contrast, the transverse processes are horizontally oriented in *Crichtonpelta*^12^, and *Ankylosaurus*^15^, and the Mongolian ankylosaurid IGM 100/1305 (cf. *Pinacosaurus*)^29,30^. The neural canal is large, wider than high, similar to that in *Crichtonpelta*^12^. The parapophysis is a subcircular protuberance, located more anteriorly relatively to the transverse process on the centrum. The centrum is a trapezoid in lateral view with the ventral edge slightly longer than the dorsal edge.

#### Dorsal vertebra

ZMNH M8960 (Fig. 4m–r) preserves one incomplete dorsal vertebra. The spool-shaped centrum has concave lateral and ventral sides and sub-circular articular faces. The articular faces are also concave in *Ankylosaurus*^15^, but almost flat in *Crichtonpelta*^12^, and *Saichania*^14^. Ventral to the diapophysis the centrum is laterally compressed. The length of the centrum is greater than the transverse width of the articular surfaces as in *Struthiosaurus*^31^. The converse is true in other ankylosaurs^7^, such as *Crichtonpelta*^12^. However, the centrum is more elongate than that in *Struthiosaurus* with a length/width ratio of approximate 1.3, greater than that of any other ankylosaurians. The neural arch is located in the anterior half of the centrum. The neural canal is relatively small compared to that in the cervicals. The canal is ovoid in cross-section, with the long axis directed vertically. The mediolaterally thin and anteroposteriorly elongate base of the neural spine extends to the posterior end of the neural arch. The plate-like transverse process is directed upward in anterior view and anterolaterally in dorsal view, the process is directed relatively lower than that in *Crichtonpelta*^12^. The diapophysis is an inverted triangle on the end of the transverse process. The parapophysis is larger than the diapophysis, sits below the transverse process has a triangular-shaped articular surface. The prezygapophyses meet ventrally form a U-shaped trough as other ankylosaurs^32^. The postzygapophyses are fused together along their lengths to form a peg-like, midline structure.

#### Sacral vertebrae

**Figure 5:**
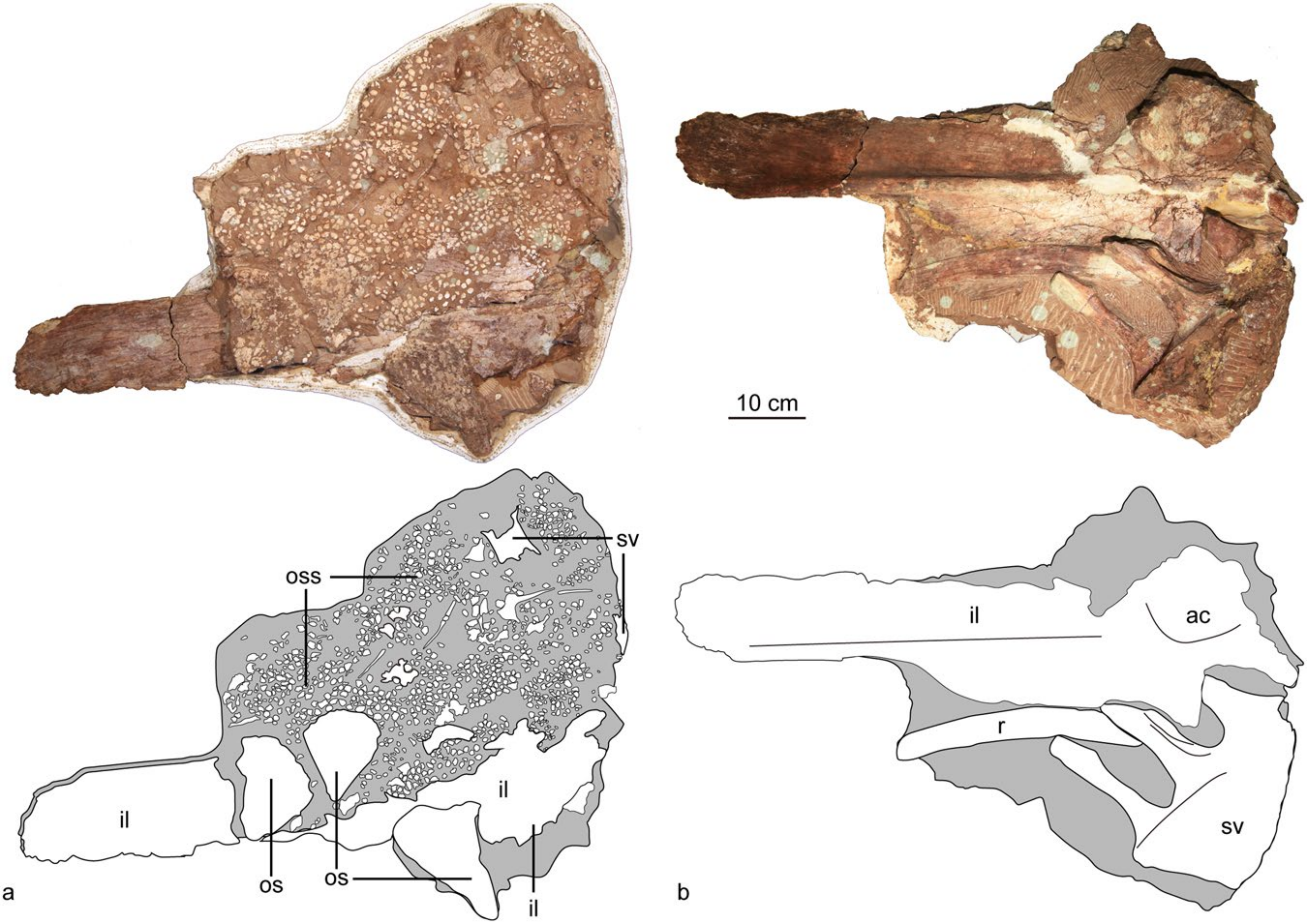
The sacral vertebrae and left ilium of *Jinyunpelta sinensis* holotype ZMNH M8960 in dorsal (**a**) and ventral (**b**) views. Abbreviations: ac, acetabulum; il, ilium; po, plate osteoderm; oss, ossicle; r, rib; sv, sacral vertebra.

Three firmly fused sacral vertebrae are preserved together with the ilium (Fig. 5). The sacral centra are broad and dorsoventrally depressed. The neural canal is well developed. Transverse processes are firmly fused with the horizontally oriented sacral ribs. The compound structure formed by the transverse processes and the sacral rib is dorsoventrally deep with an hourglass-shaped cross-section.

#### Caudal vertebrae

**Figure 6:**
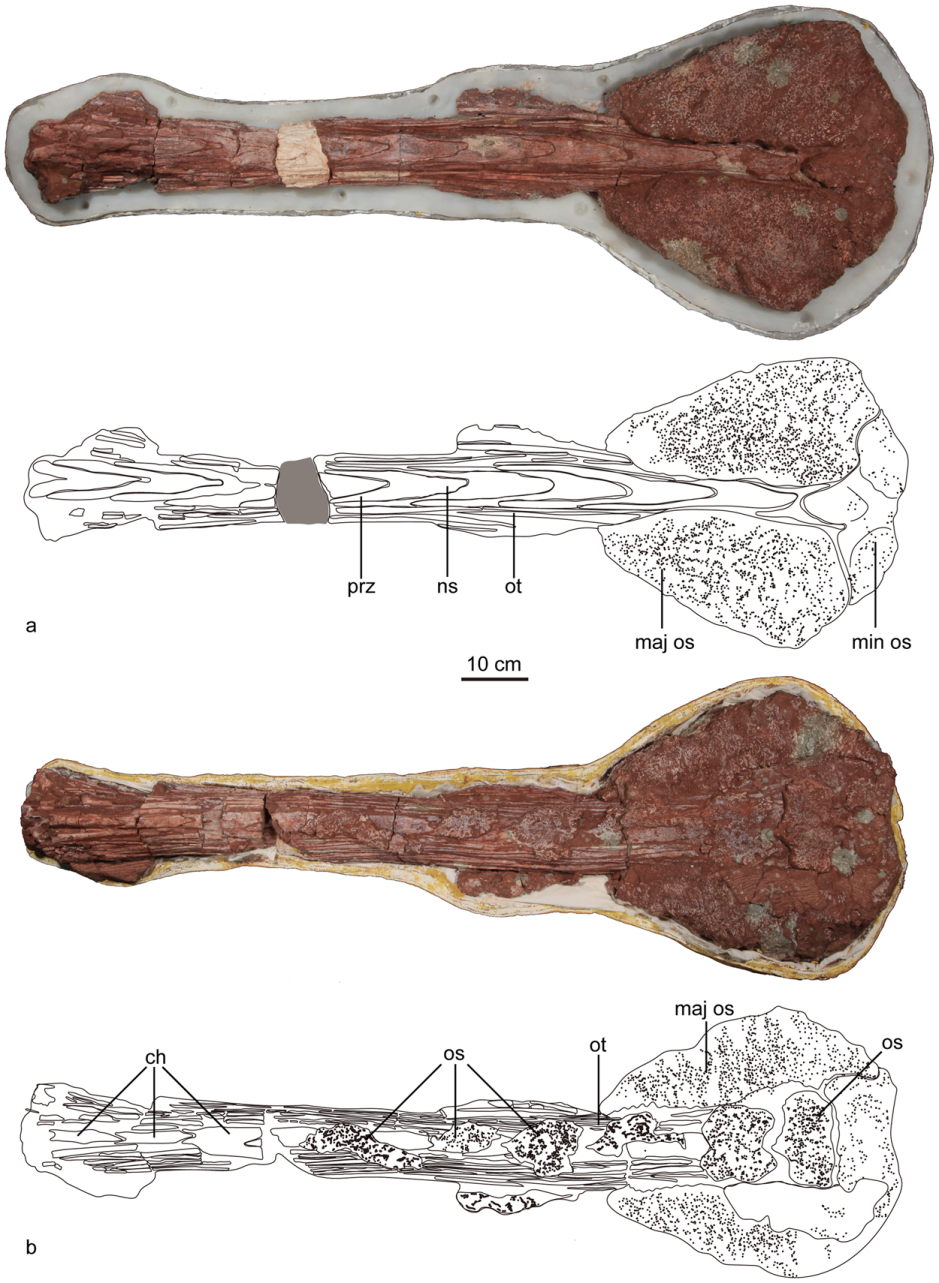
The tail club *Jinyunpelta sinensis* paratype ZMNH M8963 in dorsal (**a**) and ventral (**b**) views. Abbreviations: ch, chevron; maj os, major osteoderm of the tail club knob; min os, minor osteoderm of the tail club knob; ns, neural spine; os, osteoderm; ot, ossified tendon; prz, prezygapophyses.

Although no anterior free caudal vertebrae are preserved, the holotype includes a partial tail club knob. The paratype ZMNH M8963 includes an almost completely preserved tail club (Fig. 6, Table 3). As in other ankylosaurines, the posterior-most ten caudal vertebrae form the handle of the tail club. These vertebrae are highly modified and interlock tightly with each other to form a rigid structure^22^. These caudal vertebrae have intermediate morphology between V- and U-shaped neural spines in which the prezygapophyses diverge at an angle of approximate 14–25°. Bundles of long and parallel arranged ossified tendons are closely appressed to the lateral sides of the centra of the tail club handle (Fig. 6: ot). The tips of the ossified tendons are tapered and flattened. The chevrons are dorsoventrally short but anteroposteriorly long. They are bifurcated anteriorly, but tapers to a point posteriorly, tightly interlocking with each other along the ventral side of the vertebrae.

**Table 3:** Measurements of the tail club of *Jinyunpelta sinensis* ZMNH M8963.

Element	Measurement (cm)
Tail club length (handle+knob)	131.0
Knob maximum width	43.1
Knob maximum length	45.5
Knob maximum height	9.0
Knob width/length	0.95
Knob height/length	0.20

As in other ankylosaurines, knob osteoderms completely envelop and obscure the distalmost vertebrae^6^. The tail club knob is composed of two large lateral osteoderms, and three small medial osteoderms with one centrally located. The knob is dorsoventrally flattened rather than hemispherical. Overall, the knob has a roughly hexagonal outline in dorsal view, with the widest point close to the distal end. The knob is anteroposteriorly longer than transversely wide (45.5 cm in length and 43.1 cm in width, the width/length ratio of the tail club knob is approximately 0.95), unlike the transversely wider knobs in *Anodontosaurus*. The knob is wider than that in *Dyoplosaurus* (ROM 784) with width/height ratio 0.68^32^.

There are six osteoderms present in the ventral surface of the tail club handle (Fig. 6: os). The anterior four are incomplete, whereas the posterior two are almost complete, and wider than long with a sub-rectangular outline. Their surface pattern is similar to that of tail club knob. The ventral osteoderms are also present in Mongolian ankylosaurine and PIN 614 (cf. *Pinacosaurus*)^22^.

#### Dorsal rib

The massive dorsal ribs have well-decurved shafts relative to the rib head. As in most ankylosaurs, the dorsal surface is wide, flattened, and supported below by the deep ventral portion of the ribs, giving the rib a T-shaped cross-section proximally. Distally, this morphology becomes less distinct and eventually diminishes to an ovoid.

### Appendicular skeleton

#### Scapula

**Figure 7:**
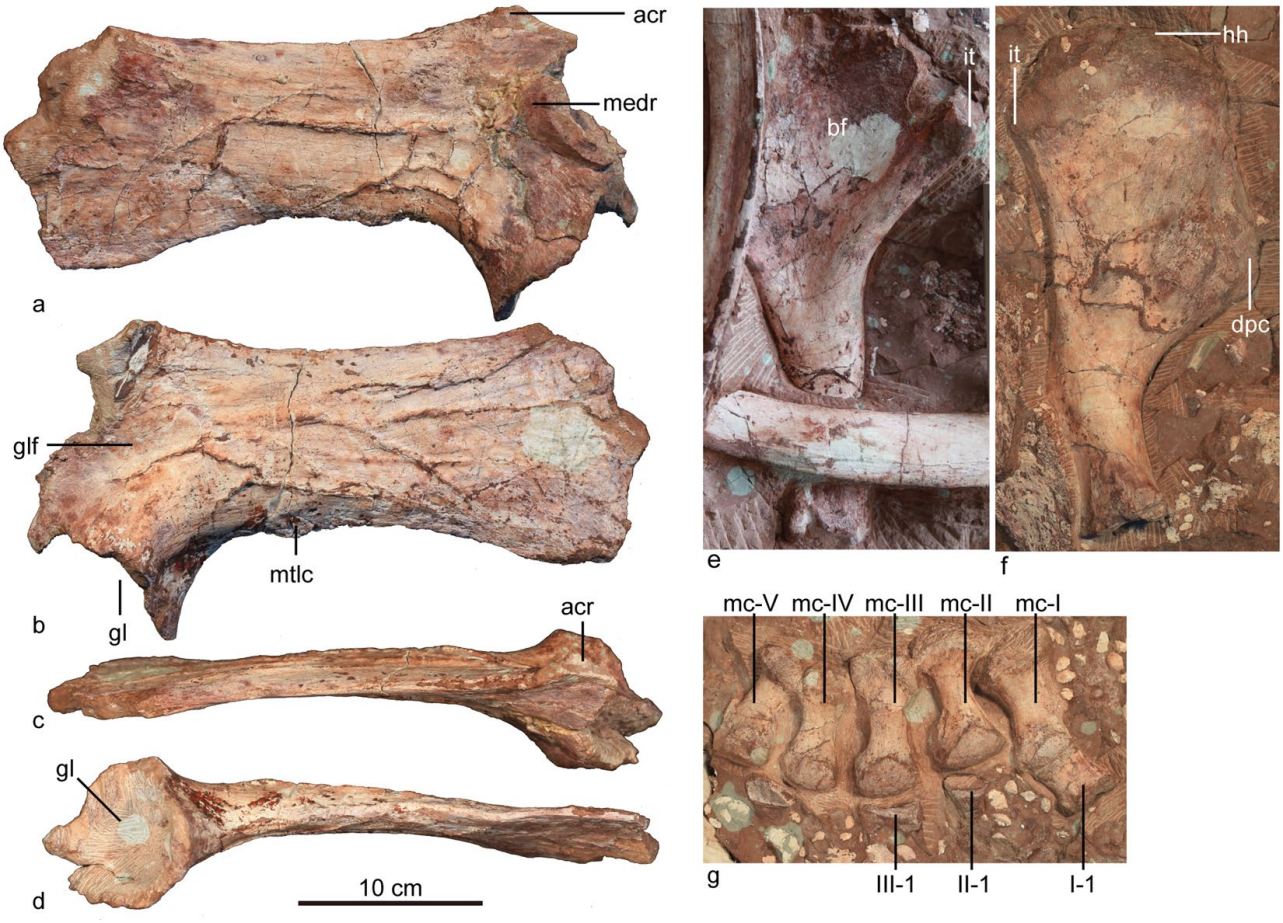
Forelimb of *Jinyunpelta sinensis* holotype ZMNH M8960. The left scapula in medial (**a**), lateral (**b**), dorsal (**c**) and ventral (**d**) views; The right humerus in anterior (**e**) and posterior (**f**) views; the right manus in dorsal view (**g**). Abbreviations: acr, acromion; bf, bicipital fossa; dpc, deltopectoral crest; gl, glenoid; glf, glenoid fossa; hh, humeral head; it, internal tuberosity; mc, metacarpal; medr, medial ridge; mtlc, enthesis of M. triceps longus caudalis.

The left scapula is preserved with both ends broken (Fig. 7a–d, Table 4). Both anterior and posterior ends are expanded, producing concave dorsal and ventral margins of the scapular blade in lateral view. The posterior expansion is stronger than in *Crichtonpelta*^12^. The incomplete acromion curves dorsolaterally. The narrowest region of the scapular blade is immediately posterior to the glenoid fossa. The dorsal margin of the scapular blade is slightly convex as in other ankylosaurs, such as *Sauropelta*, *Euoplocephalus*^7^, and *Chuanqilong*^33^. In lateral view, the anteroventral projection is very strong. There is a prominent enthesis on the ventral edge just posterior to the projection, which probably marks the insertion of the M. triceps longus caudalis, as in *Crichtonpelta*^12^, *Ankylosaurus*^15^ and *Euoplocephalus*^32^. On the medial side, the scapulocoracoid buttress is well-developed at the anterior end of the scapula.

#### Humerus

The right humerus is preserved with its distal end missing (Fig. 7e,f, Table 4). The massively built humerus bears a deltopectoral crest strongly expanded in anterior or posterior view. The surface of the deltopectoral crest has prominent striations, which may represent the attachments for the M. supracoracoideus and M. pectoralis^32^. The medial (internal) tuberosity is prominent. Anteriorly, the deltopectoral crest and humeral head bound a broad shallow fossa (bicipital fossa) with an inverted triangular outline.

**Table 4:** Measurements of the forelimb of *Jinyunpelta sinensis* holotype ZMNH M8960. Asterisk indicates incomplete measurement due to damage.

Element		Measurement (cm)
Left scapula	Midshaft dorsoventral height (minimum)	9.5
	Proximal height (maximum)	16.3*
	Distal height (maximum)	13.6*
Right humerus	Proximal mediolateral width (maximum)	15.5
	Midshaft mediolateral width (minimum)	5.4

#### Manus

The manus is pentadactyl as in *Pinacosaurus*, *Sauropelta*, and *Shamosaurus*^7^. All metacarpals are robust and expanded at the proximal and distal ends. Metacarpals become shorter and less massive moving from I to V (Fig. 7g, Table 5).

**Table 5:** Measurements of the manus of *Jinyunpelta sinensis* holotype ZMNH M8960 (cm).

Element	Length	Mediolateral width	
		Proximal	Distal
Metacarpal I	6.7	—	3.3
Metacarpal II	7.4	3.7	3.5
Metacarpal III	7.7	3.4	3.5
Metacarpal IV	6.5	3.0	3.0
Metacarpal V	6.3	3.2	2.8
Manus phalanx I-1	3.2	2.8	3.3

#### Ilium

The left ilium is preserved, with its dorsal portion obscured by the small osteoderms (ossicles) (Fig. 5a–b). The majority of the iliac blade is rotated horizontally. The preacetabular process is long, relatively straight, depressed dorsoventrally, and diverges anterolaterally from the sacrum. The dorsal surface of the preacetabular process directs dorsolaterally, which is almost directs laterally in *Crichtonpelta*^12^. A raised anterolaterally oriented ridge lies along the ventral surface of the preacetabular process, which is almost straight, however the ridge is well-curved in *Crichtonpelta*^12^. The acetabulum is imperforate, shallow and cuplike as in all ankylosaurians^7^.

A large number of small osteoderms (ossicles) are preserved together with the ilium. Most are small ossicles range from 0.5 to 2.5 cm in diameter similar to those on the ventral side of the skull. The ossicles may have occupied the space between the larger plates on the dorsal surface^15^.

Three large triangular plate osteoderms are preserved dorsal and lateral to the ilium (Fig. 6a: os). The large dorsal osteoderm is level with the small ossicles.

#### Ischium

**Figure 8:**
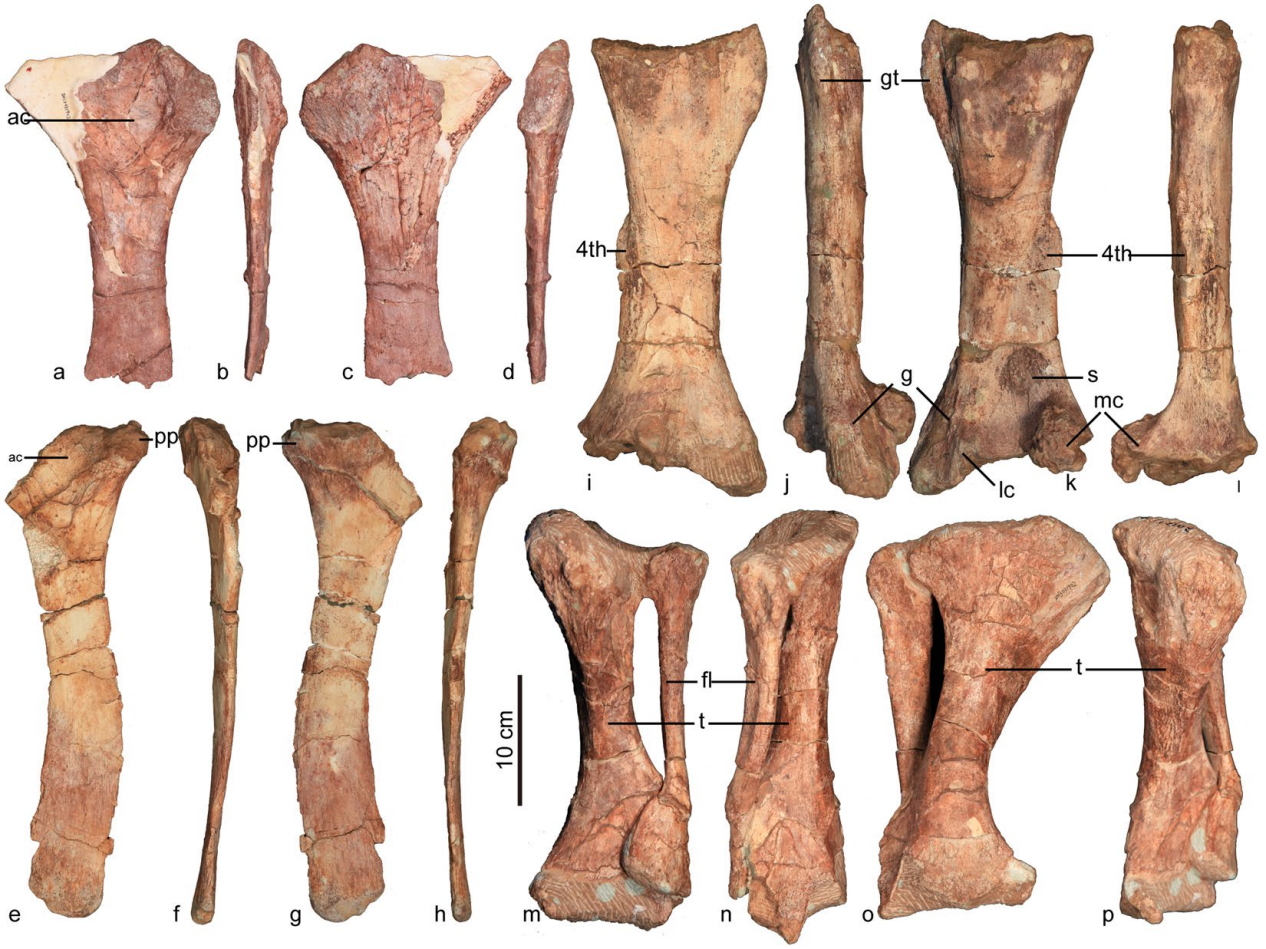
Hind limb of *Jinyunpelta sinensis*. Left ischium of ZMNH M8963 in lateral (**a**), anterior (**b**), medial (**c**) and posterior (**d**) views; the right ischium of ZMNH M8960 in lateral (**e**), anterior (**f**), medial (**g**) and posterior (**h**) views; the left femur of ZMNH M8960 in anterior (**i**), lateral (**j**), posterior (**k**) and medial (**l**) views; the left tibia and fibula of ZMNH M8963 in anterior (**m**), lateral (**n**), posterior (**o**) and medial (**p**) views. Abbreviations: 4th, fourth trochanter; ac, acetabulum; fl, fibula; g, scar for the M. gastrocnemius; gt, greater trochanter; lc, lateral condyle; mc, medial condyle; pp, pubic peduncle; s, scar; t, tibia.

Both ischia are preserved, but neither is complete (Fig. 8a–h, Table 6). The body of the ischium forms a laterally compressed shaft that abruptly terminates without a distal expansion. The ischium is wide proximally, and a sulcus on the lateral side contributes to the closed acetabulum. The ischial shaft is slightly sigmoidal in both lateral and medial views. The distal portion is curved posteriorly. The wide proximal end tapers abruptly into the ischial shaft, with the narrowest region of the shaft occurring proximally. The ischial shaft widens toward the distal end, prior to tapering again further distally, as in *Chuanqilong*^33^.

#### Femur

The right femur is preserved with both ends broken (Fig. 8i–l, Table 6). The femoral shaft is straight, massive and anteroposteriorly compressed. The proximal and distal ends are transversely expanded relative to the shaft, which has its narrowest width just proximal to the fourth trochanter. The ridge-like fourth trochanter sits on the medial margin of the femur. The trochanter is more prominent than that in *Crichtonpelta*^12^. On the distolateral surface of the shaft bears a scar for the M. gastrocnemius, which is less prominent than that in *Ankylosaurus*^15^, and *Dongyangopelta*^34^. This scar is absent in *Crichtonpelta*^12^. Mediodorsal to the medial condyle, there is a prominent scar, which is not seen in other ankylosaurians. Distally, both the medial and lateral condyles are posteriorly expanded, with the medial more prominent.

**Table 6:** Measurements of the hind limb (cm).

Element		Measurements
Left ischium ZMNH M8963	Midshaft mediolateral width (minimum)	1.7
	Midshaft anteroposterior length (minimum)	5.7
Right ischium ZMNH M8960	Midshaft mediolateral width (minimum)	1.4
	Midshaft anteroposterior length (minimum)	5.1
	Distal mediolateral width	1.7
	Distal anteroposterior length	5.8
Femur ZMNH M8960	Midshaft mediolateral width (minimum)	7.2
	Midshaft anteroposterior length (minimum)	4.5
	Proximal mediolateral width	13.3
	Distal mediolateral width (maximum)	14.9
	Midshaft Circumference	20.1
Tibia ZMNH M8963	Length	30.1
	Midshaft mediolateral width (minimum)	4.5
	Midshaft anteroposterior length (minimum)	4.6
	Midshaft Circumference (minimum)	16.0
Fibula ZMNH M8963	Length	26.7
	Proximal anteroposterior length	6.5
	Distal anteroposterior length	6.3

#### Tibia

The tibia and fibula are preserved in articulation (Fig. 8m–p, Table 6). The tibia is stout and straight. The proximal and distal ends are broadly expanded anteroposteriorly and mediolaterally, respectively, creating a twisted tibial shaft.

#### Fibula

The fibula is slender relative to the tibia (Fig. 8m–p, Table 6). It has a laterally compressed and straight shaft with rugose proximal and distal ends. The well-defined distal end indicates that the calcaneum was not fused with it.

## Methods

### Phylogenetic analysis

The systematic position of *Jinyunpelta sinensis* within Ankylosauridae was quantitatively evaluated by analyzing a recently published comprehensive dataset for the ankylosaurid phylogeny by Arbour, *et al*.^35^ with *Jinyunpelta* and *Zuul*^22^ added in. The scores for *Crichtonpelta* are revised according to Yang, *et al*.^16^, except the character 4 followed Arbour, *et al*.^35^. The revised dataset includes 59 taxa and 177 characters, and the character matrix was assembled in Mesquite version 3.2. The analysis was carried out using a traditional search with the tree bisection reconnection algorithm in TNT version 1.5. All characters were treated as unordered and of equal weight. The maximum trees in memory was set to 100000, 1000 replicates were used with 10 trees saved per replication. The analysis resulted in 100000 parsimonious trees (tree length = 566 steps, CI = 0.399, and RI = 0.667). The strict consensus tree of these 100000 MPTs lacks resolution, with nearly the whole of Ankylosauria in an unresolved polytomy (see Supplementary Fig. S1). The 50% majority rule consensus tree of 100,000 MPTs recovers the traditional ankylosaurid-nodosaurid dichotomy. *Jinyunpelta* is placed as the most basal ankylosaurine in 74% of the trees (see Supplementary Fig. S1).

**Figure 9:**
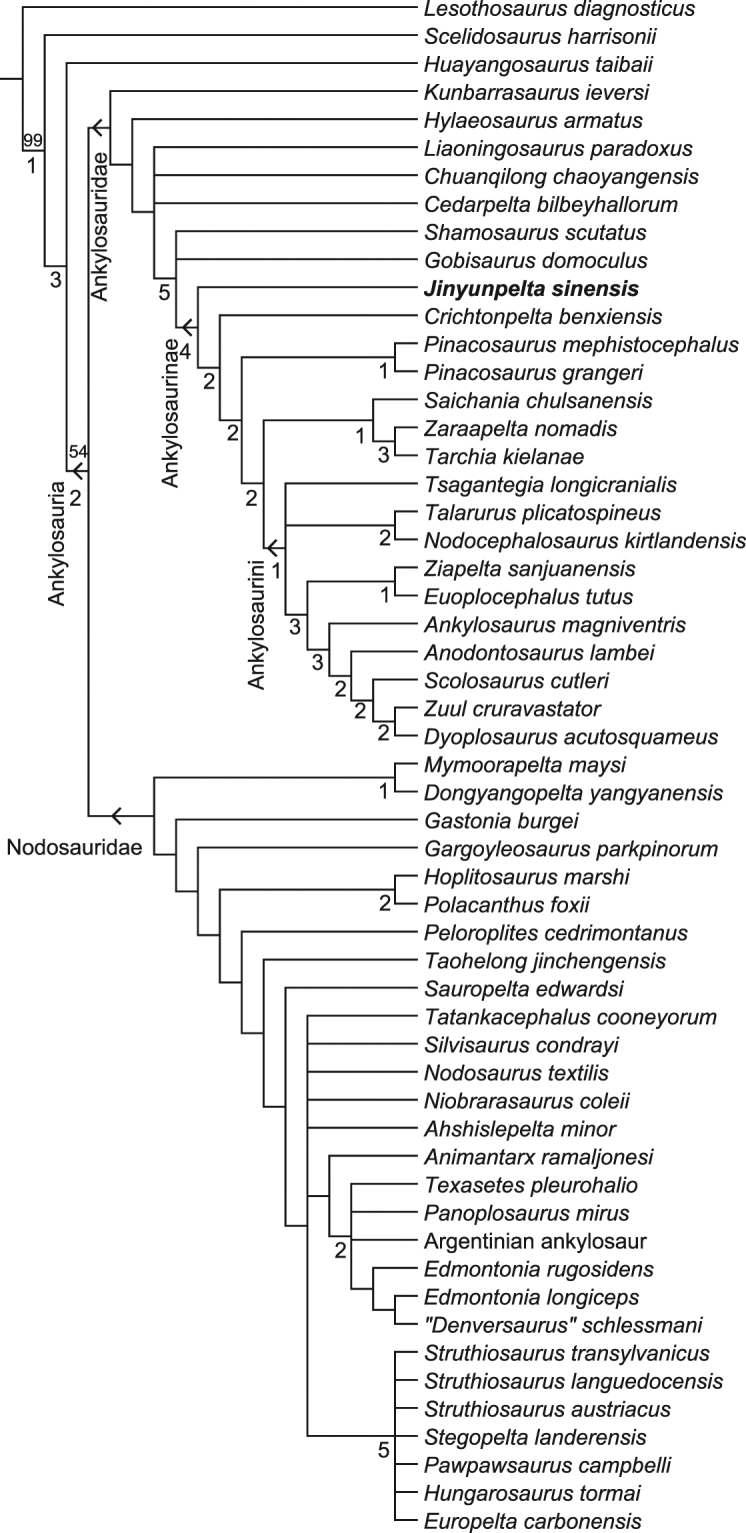
The derivative strict reduced consensus tree produced by phylogenetic analysis. *Aletopelta*, Paw Paw scuteling, *Sauroplites*, and ‘*Zhejiangosaurus*’ were pruned a posteriori to improve resolution. Values above nodes represent bootstrap proportions. Values beneath nodes indicate Bremer support.

A reduced consensus tree was calculated a posteriori which excluded four wildcard taxa (*Aletopelta*, Paw Paw scuteling, *Sauroplites*, and ‘*Zhejiangosaurus*’), and this shows considerably greater resolution (Fig. 9). *Jinyunpelta* is recovered as the most basal ankylosaurine in the derivative strict reduced consensus tree.

## Discussion

*Jinyunpelta sinensis* is identified as an ankylosaurid based on the following ankylosaurid synapomorphies: two pairs of osteodermal horns projecting from the quadratojugals and squamosals; paranasal apertures present; absence of premaxillary teeth; rugose cranial ornamentation; a well-developed tail club, with prezygapophyses and neural spines overlapping more than half the length of the adjacent vertebra, and ossified tendons in the distal part of the tail. *Jinyunpelta sinensis* also possesses several synapomorphies of Ankylosaurinae^6^: supraorbitals form a sharp, continuous edge above orbit; haemal arches fused to their respective centra; well-developed tail club knob. The overall arrangement of the cranial bones and general shape of the skull of *Jinyunpelta sinensis* is similar to that in the other basal ankylosaurids such as *Gobisaurus*, *Shamosaurus*, *Crichtonpelta*, and *Pinacosaurus*: the skull is longer than wide; the dorsal cranial ornamentation is not divided into discrete caputegulae but is instead rugose and irregular, while discrete caputegulae are present in more derived ankylosaurids like *Ankylosaurus*, *Euoplocephalus* or *Saichania*^6^. However, the premaxillary beak is relatively wider than that in *Gobisaurus*, *Shamosaurus*, and *Crichtonpelta*.

**Figure 10:**
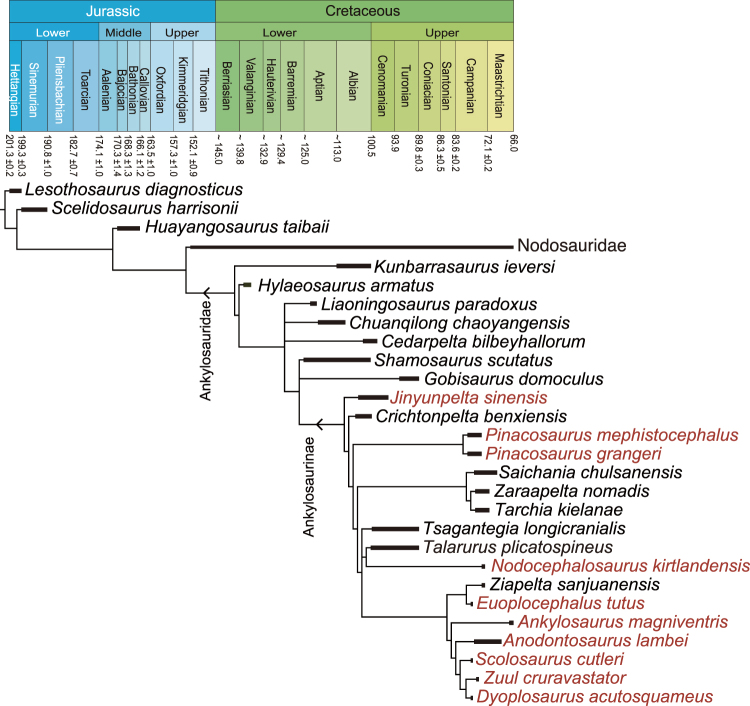
Temporal calibration of the simplified derivative strict reduced consensus tree produced by phylogenetic analysis. Taxa in red text have a tail club knob. The geologic numerical ages and coloring follow International Chronostratigraphic Chart 2017/02.

The tail club is one of the most recognizable features of derived ankylosaurids^32^. Arbour and Currie^36^ investigated the evolution of the tail club, and suggest the tail club evolved in a stepwise fashion with handle-like vertebrae preceding the distal enlarged knob. The oldest and most basal ankylosaurian known to possess the tail club knob was *Pinacosaurus* from the Campanian^36^. *Talarurus* was the oldest ankylosaurid (Cenomanian–Turonian) with a tail club but more derived than *Pinacosaurus*^36^, however, not tail club knob is preserved in *Talarurus*^37^. The tail club handle and knob are very well developed in *Jinyunpelta*. Given the uncertainty of the tail club in *Crichtonpelta*^12,16,36^, *Jinyunpelta* represents the oldest and the most basal ankylosaurian known to have possessed a tail club knob (Fig. 10). The tail club knob of *Jinyunpelta* is approximately 45.5 cm in transverse diameter, comparable with the size of the tail club from Scollard Formation (AMNH 5214, ~45 cm wide), the youngest unit preserving a tail club knob^36^. However, it is much larger than the tail club knob (MPC 100/1305, 14.6 cm wide) in the Djadokhta Formation, previously the oldest formation preserving tail club knobs^22,36^, though smaller than the largest knob from the younger formations than Djadokhta Formation (Nemegt, Dinosaur Park, and Horseshoe Canyon formations) (ZPAL MgD I/43, 62 cm wide, ROM 788, 57.2 cm wide, AMNH 5245, 59.3 cm wide^22,36^. The largest knob is approximately 44% wider than that of *Jinyunpelta*,). The relatively large size of the tail club knob of *Jinyunpelta* suggests that the large knob appeared early in ankylosaurid evolution and the size evolution of tail club is more than a simple trend of size-increase. Up to now, only *Jinyunpelta* from mid-Cretaceous possesses a well-developed tail club knob had been found, and other ankylosaur specimens with tail club knob are from Campanian or Maastrichtian. More materials from early development of the tail club knob are needed to understand the early evolution and trend of the tail club knob.

Ankylosaurian dinosaur remains are abundant in Asia, but most specimens were discovered in Mongolia or northern China. All ankylosaurians discovered from Mongolia are referable to Ankylosauridae, and except *Shamosaurus*, all belong to Ankylosaurinae^6,17^. Most ankylosaurs of China were discovered from northern China and belong to ankylosaurids, such as *Liaoningosaurus*, *Chuanqilong* and *Crichtonpelta* from Liaoning^12,16,33,38^, *Gobisaurus* from Nei Mongol and Henan^6,9,10^, *Pinacosaurus* from Nei Mongol and Shandong^39–41^, and *Saichania* from Shanxi^6,42,43^. *Jinyunpelta* therefore represents the southernmost occurrence of definitive ankylosaurid remains from Asia.

Two ankylosaurians, ‘*Zhejiangosaurus*’^44^ and *Dongyangopelta*^34^, had been reported from Zhejiang Province. *Dongyangopelta* was recovered as Nodosauridae^6,34^. Althought ‘*Zhejiangosaurus*’ was recovered as Nodosauridae^45^, it was recovered as Ankylosauridae later and treated as *nomen dubium* in latest phylogenetic analysis^6^. *Jinyunpelta* therefore represents the first definitive and diagnosable ankylosaurid dinosaur and first ankylosaurine dinosaur from Zhejiang Province. The new discovery suggests that both nodosaurid and ankylosaurid dinosaurs inhabited Zhejiang Province and presents an important addition to the known diversity and palaeogeographical distribution of ankylosaurians in Asia.

### Data Availability

The high-resolution photos of the skull are available in the figshare repository, 10.6084/m9.figshare.5895958.

## Conclusions

*Jinyunpelta sinensis*, gen. et sp. nov. is a new basal ankylosaurine dinosaur based on two specimens with an almost complete skull, an almost complete tail club, and postcranial remains. *Jinyunpelta* represents the oldest and the most basal ankylosaurian known to have a well-developed tail club knob. It demonstrates that a large and highly modified tail club knob evolved at the base of the ankylosaurine at least about 100 million years ago. *Jinyunpelta* represents the first definitive ankylosaurid dinosaur from southern China, and expands the known diversity and palaeogeographical distribution of ankylosaurians in Asia.

## Electronic supplementary material

Supplementary Information

## References

[1] Bureau of Geology and Mineral Resources of Zhejiang Province. *Regional Geology of Zhejiang Province*. (Geological Publishing House, 1989).

[2] Zheng W, Jin X, Shibata M, Azuma Y, Yu F (2012). A new ornithischian dinosaur from the Cretaceous Liangtoutang Formation of Tiantai, Zhejiang Province, China. Cretaceous Research.

[3] Wang, Y.-Z., Jiang, Y.-G. & Chen, K.-Q. The study of dinosaur fossil and Late Cretaceous basin of Tiantai in Zhejiang. *Unpublished file report of the Hydrology and Engineering Geological Brigade of Zhejiang Province and Institute of Mineral Resources Chinese Academy of Geological Sciences* (2000).

[4] He H (2013). SIMS zircon U–Pb dating of the Late Cretaceous dinosaur egg-bearing red deposits in the Tiantai Basin, southeastern China. Journal of Asian Earth Sciences.

[5] Jiang Y, Qian M-P, Xing G-F, Jiang Y-G (2016). Zircon U-Pb Age of the dinosaur-bearing strata in the Tiantai Basin of Zhejiang. Journal of Stratigraphy.

[6] Arbour VM, Currie PJ (2016). Systematics, phylogeny and palaeobiogeography of the ankylosaurid dinosaurs. Journal of Systematic Palaeontology.

[7] Vickaryous, M. K., Maryańska, T. & Weishampel, D. B. In *The Dinosauria* (eds David B. Weishampel, Peter Dodson, & Halszka Osmólska) Ch. 17, 363–392 (University of California Press, 2004).

[8] Leahey LG, Molnar RE, Carpenter K, Witmer LM, Salisbury SW (2015). Cranial osteology of the ankylosaurian dinosaur formerly known as *Minmi* sp. (Ornithischia: Thyreophora) from the Lower Cretaceous Allaru Mudstone of Richmond, Queensland, Australia. PeerJ.

[9] Xu L (2007). New nodosaurid ankylosaur from the Cretaceous of Ruyang, Henan Province. Acta Geologica Sinica.

[10] Vickaryous MK, Russell AP, Currie PJ, Zhao X-J (2001). A new ankylosaurid (Dinosauria: Ankylosauria) from the Lower Cretaceous of China, with comments on ankylosaurian relationships. Canadian Journal of Earth Sciences.

[11] Burns ME, Currie PJ, Sissons RL, Arbour VM (2011). Juvenile specimens of *Pinacosaurus grangeri* Gilmore, 1933 (Ornithischia: Ankylosauria) from the Late Cretaceous of China, with comments on the specific taxonomy of *Pinacosaurus*. Cretaceous Research.

[12] Lü J, Ji Q, Gao Y, Li Z (2007). A new species of the ankylosaurid dinosaur *Crichtonsaurus* (Ankylosauridae: Ankylosauria) from the Cretaceous of Liaoning Province, China. Acta Geologica Sinica - English Edition.

[13] Hill RV, Witmer LM, Norell MA (2003). A New Specimen of *Pinacosaurus grangeri* (Dinosauria: Ornithischia) from the Late Cretaceous of Mongolia: Ontogeny and Phylogeny of Ankylosaurs. Am. Mus. Novit..

[14] Maryańska T (1977). Ankylosauridae (Dinosauria) from Mongolia. Palaeontologica Polonica.

[15] Carpenter K (2004). Redescription of *Ankylosaurus magniventris* Brown 1908 (Ankylosauridae) from the Upper Cretaceous of the Western Interior of North America. Canadian Journal of Earth Sciences.

[16] Yang J, You H, Xie L, Zhou H (2017). A new specimen of *Crichtonpelta benxiensis* (Dinosauria: Ankylosaurinae) from the Mid-Cretaceous of Liaoning Province, China. Acta Geologica Sinica (English Edition).

[17] Arbour VM, Currie PJ, Badamgarav D (2014). The ankylosaurid dinosaurs of the Upper Cretaceous Baruungoyot and Nemegt formations of Mongolia. Zoological Journal of the Linnean Society.

[18] Gilmore, C. W. Two new dinosaurian reptiles from Mongolia with notes on some fragmentary specimens. *American Museum Novitates*, 1–20 (1933).

[19] Vickaryous MK, Russell AP (2003). A redescription of the skull of *Euoplocephalus tutus* (Archosauria: Ornithischia): a foundation for comparative and systematic studies of ankylosaurian dinosaurs. Zool. J. Linn. Soc..

[20] Tumanova TA (1983). The first ankylosaur from the Lower Cretaceous of Mongolia. Trudy Sovm. Sov.-Mong. Paleontol. Eksped.

[21] Molnar, R. E. In *Proceedings of the Gondwanan Dinosaur Symposium. Memoirs of the Queensland Museum* (eds F. E. Novas & R. E. Molnar) 653–668.

[22] Arbour VM, Evans DC (2017). A new ankylosaurine dinosaur from the Judith River Formation of Montana, USA, based on an exceptional skeleton with soft tissue preservation. Royal Society Open Science.

[23] Arbour VM (2014). A new ankylosaurid dinosaur from the Upper Cretaceous (Kirtlandian) of New Mexico with implications for ankylosaurid diversity in the Upper Cretaceous of Western North America. PLoS ONE.

[24] Arbour VM, Mallon JC (2017). Unusual cranial and postcranial anatomy in the archetypal ankylosaur Ankylosaurus magniventris. FACETS.

[25] Sereno PC (1999). The evolution of dinosaurs. Science.

[26] Miles CA, Miles CJ (2009). Skull of *Minotaurasaurus ramachandrani*, a new Cretaceous ankylosaur from the Gobi Desert. Current Science.

[27] Lambe LM (1919). Description of a new genus and species (Panoplosaurus mirus) of armored dinosaur from the Belly River Beds of Alberta. Transactions of the Royal Society of Canada.

[28] Vickaryous MK (2006). New information on the cranial anatomy of Edmontonia rugosidens Gilmore, a Late Cretaceous nodosaurid dinosaur from Dinosaur Provincial Park, Alberta. J. Vert. Paleontol..

[29] Carpenter K (2011). *Saichania chulsanensis* (Ornithischia, Ankylosauridae) from the Upper Cretaceous of Mongolia. Palaeontographica, Abt. A: Palaeozoology-Stratigraphy.

[30] Arbour VM, Currie PJ (2013). The taxonomic identity of a nearly complete ankylosaurid dinosaur skeleton from the Gobi Desert of Mongolia. Cretaceous Research.

[31] Pereda-Suberbiola, X. & Galton, P. M. In *The Armored Dinosaurs* (ed Kenneth Carpenter) 173–210 (Indiana University Press, 2001).

[32] Arbour VM, Currie PJ (2013). *Euoplocephalus tutus* and the diversity of ankylosaurid dinosaurs in the Late Cretaceous of Alberta, Canada, and Montana, USA. PLoS ONE.

[33] Han F, Zheng W, Hu D, Xu X, Barrett PM (2014). A new basal ankylosaurid (Dinosauria: Ornithischia) from the Lower Cretaceous Jiufotang Formation of Liaoning Province, China. PLoS ONE.

[34] Chen R (2013). A new nodosaurid ankylosaur from the Chaochuan Formation of Dongyang, Zhejiang Province, China. Acta Geologica Sinica (English Edition).

[35] Arbour VM, Zanno LE, Gates T (2016). Ankylosaurian dinosaur palaeoenvironmental associations were influenced by extirpation, sea-level fluctuation, and geodispersal. Palaeogeography, Palaeoclimatology, Palaeoecology.

[36] Arbour VM, Currie PJ (2015). Ankylosaurid dinosaur tail clubs evolved through stepwise acquisition of key features. J Anat.

[38] Xu X, Wang X-L, You H-L (2001). A juvenile ankylosaur from China. Naturwissenschaften.

[37] Maleev, E. A. A new family of armored dinosaurs from the Upper Cretaceous of Mongolia. Dokl. Akad. Nauk S.S.S.R. 87, 131–134 (1952).

[39] Young, C. C. *On a new nodosaurid from Ninghsia*. Vol. 11 (Geological Survey of China, 1935).

[40] Buffetaut E (1995). An ankylosaurid dinosaur from the Upper Cretaceous of Shandong (China). Geological Magazine.

[41] Godefroit P, Pereda-Suberbiola X, Li H, Dong Z-M (1999). A new species of the ankylosaurid dinosaur *Pinacosaurus* from the Late Cretaceous of Inner Mongolia (P. R. China). Bull. Inst. Roy. Sci. Nat. Belg. Sci. Terre.

[42] Pang Q-Q, Cheng Z-W (1998). A new ankylosaur of the Late Cretaceous Tianzhen, Shanxi. Progress in Natural Science.

[43] Barrett PM, You H-L, Upchurch P, Burton AC (1998). A new ankylosaurian dinosaur (Ornithischia: Ankylosauria) from the Upper Cretaceous of Shanxi Province, People’s Republic of China. Journal of Vertebrate Paleontology.

[44] Lü J (2007). New nodosaurid dinosaur from the Late Cretaceous of Lishui, Zhejiang Province, China. Acta Geologica Sinica (English Edition).

[45] Thompson RS, Parish JC, Maidment SCR, Barrett PM (2012). Phylogeny of the ankylosaurian dinosaurs (Ornithischia: Thyreophora). Journal of Systematic Palaeontology.

[46] Jin, X. *et al*. *Dinosaurs walking in Zhejiang: dinosaurs of Zhejiang Province*. 143 (Zhejiang People’s Finearts Publishing House, 2012).

[47] Lawver DR, Jin X, Jackson FD, Wang Q (2016). An avian egg from the Lower Cretaceous (Albian) Liangtoutang Formation of Zhejiang Province, China. Journal of Vertebrate Paleontology.

