# Supplementary material for: The most basal ankylosaurine dinosaur from the Albian–Cenomanian of China, with implications for the evolution of the tail club
Source: Sci Rep. 2018 Feb 27;8:3711. doi: 10.1038/s41598-018-21924-7 (PMC5829254; doi:10.1038/s41598-018-21924-7)
Supplement: Supplementary file 1 — Supplementary Information [file 41598_2018_21924_MOESM1_ESM.pdf]

Supplementary Information:

The most basal ankylosaurine dinosaur from the Albian–Cenomanian of China, with implications for the evolution of the tail club

Wenjie Zheng[1, 2, 3, 4, 5], Xingsheng Jin[1], Yoichi Azuma[1, 6, 7], Qiongying Wang[8], Kazunori Miyata[6, 7], Xing Xu[2, 3]

1. Zhejiang Museum of Natural History, Hangzhou, Zhejiang 310014, People's Republic of China.
2. Key Laboratory of Vertebrate Evolution and Human Origins of Chinese Academy of Sciences, Institute of Vertebrate Paleontology and Paleoanthropology, Chinese Academy of Sciences, Beijing 100044, People's Republic of China.
3. CAS Center for Excellence in Life and Paleoenvironment, Beijing, 100044, People's Republic of China
4. University of Chinese Academy of Sciences, Beijing 100049, People's Republic of China.
5. State Key Laboratory of Palaeobiology and Stratigraphy (Nanjing Institute of Geology and Palaeontology, CAS, People's Republic of China).
6. Fukui Prefectural Dinosaur Museum, Katsuyama, Fukui 911-8601, Japan.
7. Fukui Prefectural Dinosaur Museum, Katsuyama, Fukui 911-8601, Japan.
8. Jinyun Museum, Jinyun 321400, People's Republic of China.

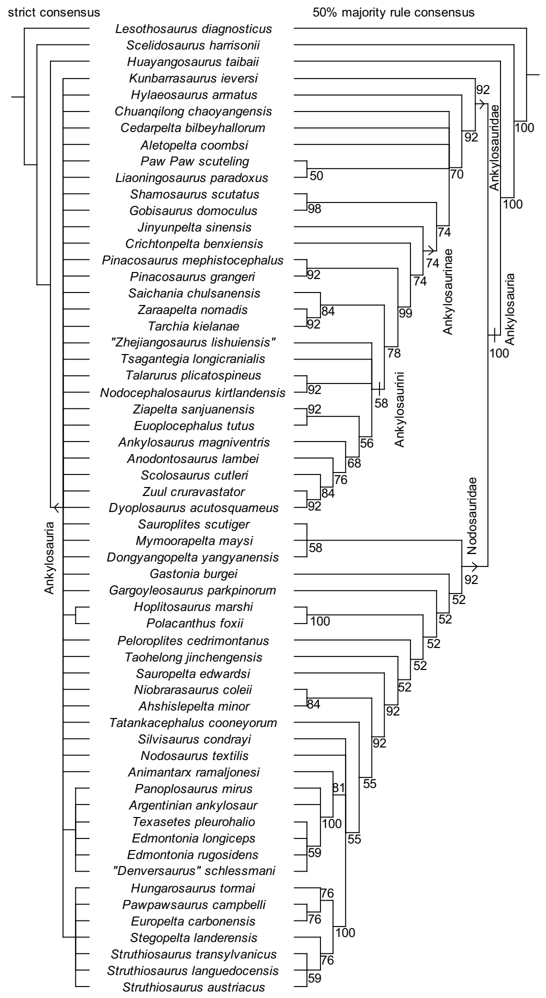

Figure S1. Results of the phylogenetic analysis, showing the strict consensus, 50% majority rule, Frequencies are indicated on the 50% majority rule tree.

Text S1. Data matrix in TNT format.

xread
177 59
Lesothosaurus_diagnosticus
000000???0000000???0000000000000000000000000000000000000?00000000
00000000?0000001?000000000000000000000?0?0000000000000000?0000?000000
00000000100?00001000000000000000000000000
Scelidosaurus_harrisonii
000000???0?00000???000000000010?000000000000110000000000000?10000000
00??100100001?1100?0010000000101100000??00?00000000?0000000?0010?0000001
1000??0000000001011[1 2]11110????100010121200
Huayangosaurus_taibaii
000000000000000000?000000000010??0000?000001000000000000000??11100000
000010010?000111?00001001???11111?000001100000?0001000000?101000000000
10?0???2010100021221?????0000000000110100
Ahshislepelta_minor
??
??001111?110??????????????0001??????????
?????22121???????1??????????

Aletopelta_coombsi
???
?????????????111?????????????0???0?0???????????????1??????0?????????????11
11??1?2??????2????1???3?1???
Animantarx_ramaljonesi
011?1??????????????????????????????11??11?0011??0?0001101?111???????1
0110??1111110????1?0????111111?1?????????1001111210 1?1111?01111011011????
??110????????????????????????????
Ankylosaurus_magniventris
11110100?11?112101?2310101110111011?210?1110121201021111111100001
111111110111100 1?211111???1111111??000011311???01110010????0?????0110100
???2010111???122?????22100??????1??12
Anodontosaurus_lambei
11110100?1111110?1231010111111101112101111012121102111111111000011
1111111101111001?21111?????????1110?00?11111????????????1110011?101001????
??01011112?122??????220??????????23
Argentinian_ankylosaur
???
??????????????????????01111?1012?0????????????????????????????????????111110
????1?1??????1??????????????
Cedarpelta_bilbeyhallorum
11100?00?01?0?0????10000?00?100?1000?1?0??1?0????000??101?11110??0???
01?1111011??1????00??????1??????1100??0?0???11?????????????????011010????
10101????????????????????????????
Chuanqilong_chaoyangensis
?1?1???101?111????????????
?????1111?0???111????1??101???????000?1????0100001?11100011?0000100??2211
?11102??2?????0?????0????????00
Crichtonpelta_benxiensis
111101???????????1?1000000011????000?211011?012110101111 10???11000?1?
011111??????????????????1101111 0010?0????00101111010?11100?1?1??0000???
?20101110??????????????????????????
"Denversaurus"_schlessmani
01101?0??120??00???2110100??1????0???11???1?001110??0?????1?00????????
111???
????????????????????????????
Dongyangopelta_yangyanensis
???
????????????????????????11110?????????????????????????????????????1???????
???122????????????12??2?????
Dyoplosaurus_acutosquameus
??1?????????????????2?10????????????????11?1???0?????1021??????????????????
????????????????1???????????????0100111?1???????????????110011?101????0??2201?
111121122?????????1111??12211
Edmontonia_rugosidens
01101000?12001000??2110100111110?0???111??1?0011100100???11?1?11???
01111101?1111100?011110?111?0111111??2?1??0?????????????????????1????????
???????????121?????3102????????????
Edmontonia_longiceps
11101000?12001000??2110100111110?01??11???110011100100?11?11111110??

?11111????111100?011?1????????????11??????????????????????11111?1011??11????
????1?????111??????10????????1?????

Euoplocephalus_tutus

1111010011111111011231010111111110111211111101212010211111111000011
11111111011111011211110??0111111110000011111011011100101111001111010010
01?1201011112112221120221 0?111??12?12

Europelta_carbonensis

0?101???????????????2110????11?0??0???110????0111100101101 11???01??????
101?1???101???10?1100??01?0101111002?100??0?0?1??????0??????1?00111?????1
?1101011??122?????????????11?03???00

Gargoyleosaurus_parkpinorum

0110000001110000000?221011000101??001?210??11012110000012?0111100?01
0?0100101011111110??000010101????1???0?0????????????????????????????0????????
??211001????122221??2100??10202?2???

Gobisaurus_domoculus

11100110?01101010??100000000101?1000?110?11101212010011 1?011110000?
?00110100?1????????1????????????11?????????11?????????????????????0????0?0000???
1??????????????????????0?????????00

Hungarosaurus_tormai

0?????01?0?10000?????????????1?0???????????1?0??111?????0???101??0??????1
?1????1101110??0?00?1??11111111110001000?00111012001?1?10??1010?11000??1
211010????12???????10???11??0???00

Hylaeosaurus_armatus

???1???????????????????????????
??????????????????????01??00?0????0100011100?0100000??111??0??????0?????????
??00??111???2??????????2??12?0?

Liaoningosaurus_paradoxus

??
??????????????0111???????????0??0???01010????0?0??00?1110001100 10000?102?00
?111000??????????????????????????

Kunbarrasaurus_ieversi

111000????????0????2???????1101?10???210??1??????00???00111111 0???1??0
1001010?1?11?????11?0??01??????10?????0??1??????????????0010???0?00?000?????
?????????121????11100111 02?1212??

Mymoorapelta_maysi

??
?????????????????????????11010?010?10010?????????????1011010?01?00010??1????
??????122?????????????122?2?2?00

Niobrarasaurus_coleii

?1???????????????????1000??????1????0???1?????????11??????????????????????????
?????????????????111????1?1???111111 0000??????1????????1110??101??00011??2111
010111111 2???????????010??????00

Nodocephalosaurus_kirtlandensis

111????????????????2?21???1?1???1011?22????1012120?0??1??11????????????1
?111??????????????????????????????0???
??????2222120????0???????????

Nodosaurus_textilis

??
???????????????????????????111111???000?0??????????????111??1101??????????1111?1?
011?12????2??????000?0312???

Panoplosaurus_mirus
01101100???0??000??21101101?1???101??110??1?0011100100?010111111111??
01111101?1111?101211???0111????1?11?????????10001102001????????????1101?1
?1???????1??121????12100????????????
Paw_Paw_scuteling
?????????????????????????11?????????????????????????0?0?1?????????????1
1?????????????111???01?11?????????0????000?0??21???1?100??????0000??????0?1
????????????????????????????
Pawpawsaurus_campbelli
01101000?02?00010??2221110211000101 1?1101111011110010011001111111 1
01110110101????????01???
?????????????????????0???????????
Peloroplites_cedrimontanus
0?101?00?111???????2?10??????????????110?01?00111000 0110?0101?0???????
11?1???0???000??1000???01?10011?1012?0?????1??1112101?111000111????????01
10111000??????????????????????????
Pinacosaurus_grangeri
11110100111110101?01000200?1111011?2022101100121101??1111111100001
1101101110111110011111 10??01?11111100?0011111010011?10?01111001111 01001
0010120101111 21122????02210101020?12212
Pinacosaurus_mephistocephalus
11110100??????101???0?010???11???0?1?211?1100222101??1?0??????????????
????????1?10???1111101????????11????011111????1111??0?11100????????10?10???
????????122????022100???1???????
Polacanthus_foxii
???1???211????????????????????????
????????????0??????01??1?01?110101000?1100?11??????1010001011????????1211
01011??122100???101?0202021??00
Hoplitosaurus_marshi
??
??????????????????????????????????????1000110200???????????????10???21011
0?1??121??????????020??2??????
Gastonia_burgei
01100101?11?11000??1000000001110 1000?110?1110121100000111001110000 1
?0111010001????????1000?????????1111010?0001?????11020?1?11100010010100?
0???201010????1222 1121?2?0?120202?2100
Saichania_chulsanensis
1111000111111111??2222110111011101112110111012121101111111111100011
10111111111111011211110111111??11?????????????1??1?110?01???????????1010010
1??????????122????0222000??10??????
Sauropelta_edwardsi
011010?????????????????0??????0??10???11001110111101?0001101111???11??
1001?101111110?1?1101110?0110101111000001010001112101011101010110100111
021110100111122????13201?01130111100
Sauroplites_scutiger
??
??????????????????????????1???
???1?2?????????????12??2??????
Scolosaurus_cutleri
11110??????????1??1?23101?1111????011?211??1101212110211111111110???????

111111???????????111???????1???100?0011111011?1110??0?1110011010?001????1?
???11?121122?????2210111110112212
Shamosaurus_scutatus
11100110?011?1010??100000000101?1000?110?11101212010111010111110011
?0011011??1111000?11??
??????????1???????2210????????????
Silvisaurus_condrayi
01101000?02000000??21101???0100?10???110?11?0011100?00011011111?11?
01101101011111????01111???111111010?1??0???????????????0??????????????????
??????????122?????3101????????????
Stegopelta_landerensis
??
??????????????1?????00?1?1???????????????????20?1????????????????1?????????
?????????????10??????13?????
Struthiosaurus_austriacus
0?1?1???????????????2?10???????????????11?????0?????01100?0?1???????????1
1110?0?111????0?1??????0?10?111?1???1????????11020?0?11100?1??0????????121
1010????121?????3101??00????????
Struthiosaurus_languedocensis
??
??????????????111??????10111111???1000??????????????111?1?1000??????????????
?????121?????????????10????????
Struthiosaurus_transylvanicus
0?1?0???????????????2?1???????????????????1????0?????0101????????1???????11
11???????????????????000????1????????????????11120?0?????????????????????????
????????????????????????????
Talarurus_plicatospineus
111100????????????102?21??0???????0?1?210?1?1012??01021?1??????0???????
1111110???????????????????11?0111000001111101101111000?1110011110100100 10
120101111??12???????2?0?????0??????
Taohelong_jinchengensis
??
?????????????????????????1????10???????????????????1?1?1?10?????????????????
????12??????????????12??2?????
Tarchia_kielanae
11110001111?1?111??222212021111?1122?221?110122221101010101111 0000?
0101111111111110112111110???
?????????????????????????????????
Tatankacephalus_cooneyorum
0?101????????1??????2110????0111??????11??1?101111?0?00?1??????1?01?1?0
?010010?????????0111??
?????????2?????????????????????
Texasetes_pleurohalio
??
??????????????111????1???????1????0????1001111210??????????????0?111???1101?
?01??????????????????????????
Tsagantegia_longicranialis
11100000?11111100??22101001111101011?210?1110121?010111111111110001?
001111?10?????????11??0??
???????????????????????????????

Zaraapelta_nomadis
111?00???????????02?22???2?1???1?221220?1110121211000111111111001?1?
?1111110??
??????????????????????????????
"Zhejiangosaurus_lishuiensis"
??
??????????????????????1???1101?001???????????????11100?111?1??????121101
1?12??????????????????????????
Ziapelta_sanjuanensis
1111010??11??1110??2310101111111??011?211?11101???01021??????1?0?0?1??
?1111??0?????????1???
?????????2??????220???????????
Zuul_cruravastator
11110100?110?1110??2310101111111?1012?21111110121211021111111100001?
00111111?1111000?001000??0???1?1??0?0?011111????????????1??0011???????????
???????????122????1?????1112??12211
Jinyunpelta_sinensis
1110000111111??011??1000???011????000?21?0??????1?0????????????????????
????????1????0101??????01??1?11??????0?1?1??????1??1????100?1??0?00?1?101?
???1?????????????????1????1???11
;

proc /;
comments 0
;
